# Root decomposition affects soil hydraulic properties in four contrasting herbaceous species

**DOI:** 10.1007/s11104-026-08331-y

**Published:** 2026-02-16

**Authors:** D. Boldrin, A. K. Leung, M. Marin, J. A. Knappett, A. G. Bengough, K. W. Loades

**Affiliations:** 1 Department of Ecological Sciences, The James Hutton Institute, Invergowrie, Dundee, DD2 5 UK; 2 State Key Laboratory for Climate Resilience of Costal Cities, Department of Civil and Environmental Engineering, The Hong Kong University of Science and Technology, Clear Water Bay, Hong Kong SAR, China, Hong Kong; 3 School of Biological Sciences, University of Aberdeen, Aberdeen, AB24 3UU UK; 4 Department of Engineering Science, University of Oxford, (formerly University of Dundee), Oxford, OX1 3PJ UK; 5 School of Science and Engineering, University of Dundee, Dundee, DD1 4HN UK

**Keywords:** Bio-pores, Decomposition, Plant-soil interactions, Root channels, Soil structure

## Abstract

**Background and aims:**

To optimise soil conditions for agriculture and engineering, we must better understand how root growth and decomposition affect soil hydraulic properties. This paper investigates soil hydraulic properties down soil columns containing grass, forb, and legume species before and after root decomposition.

**Methods:**

Contrasting species (2 grasses, 1 forb, 1 legume) were grown within repacked soil columns. These soil columns were divided horizontally into 60 mm cores and hydraulic conductivity (*K*_*s*_) was measured both before and after 7-month incubation of cores at either 5 °C or in a heated glasshouse (18–25 °C). Water sorptivity, hydrophobicity and hardness were measured in root-channel walls after decomposition in cores divided longitudinally. Soil water-release characteristics were measured in small cores sampled down the soil profile.

**Results:**

Vegetated soil averaged up to 5.6-fold greater *K*_*s*_ than fallow soils, varying greatly between species. *K*_*s*_ decreased rapidly down the columns in fallow soil, whilst *D. carota* and *L. corniculatus* soils had more uniform *K*_*s*_ with depth. The soil of root-channel walls showed distinct sorptivity and hydrophobicity compared to control bulk-soil.

**Conclusion:**

Appropriate species choice can increase *K*_*s*_. Roots and their decomposition greatly affect these soil physical properties down the profile, influencing water dynamics in plant communities and soil-mediated ecosystem services.

**Supplementary information:**

The online version contains supplementary material available at 10.1007/s11104-026-08331-y.

## Introduction

Rooted soils represent one of the most hydrologically active regions of the pedosphere (Bengough 2012), and are a key component of water and climate regulation (Lee et al. 2005). In addition to the well-recognised role of plant roots in water uptake and redistribution, as well as evapotranspiration (Oki and Kanae 2006; Prieto et al. 2012), roots can actively engineer soil inducing changes in soil structure and soil hydrological properties (Ahmadi et al. 2017; Boldrin et al. 2022; Jin et al. 2017; Lu et al. 2020). The role of plant roots in determining soil characteristics and properties is such that roots are not just “in” soil but an integral part of it, given their major role in soil formation and function (Gregory 2022). These “root-influenced properties” include soil water retention and hydraulic conductivity (Leung et al. 2018, 2023; Ni et al. 2019; Vergani and Graf 2016; Whalley 2005).

Plant roots can affect soil structure and properties through a variety of chemical and physical processes including the exudation of hydrophilic or hydrophobic compounds, direct penetration, radial expansion and water extraction. These processes, and the associated modifications of soil hydrological properties, are highly dynamic (Carminati et al. 2010; Carminati and Vetterlein 2013), and range from rhizosphere (few millimetres (Bengough 2012)) to ecosystem (Archer et al. 2015) scale. Most of the physical processes associated with soil hydrology act on soil pores and can result in the alteration of water retention or conductivity according to the pore diameter-class influenced by the processes. Whilst large continuous pores (i.e., macropores; > 30 μm) are major drivers of saturated hydraulic conductivity and drainage, smaller pores are responsible for water retention (Greenland 1981). For instance, Bodner et al. (2014) reported that coarse roots enhanced microporosity by 30%, and a dense fibrous root system induced a heterogenization of pore spaces.

In general, water in soil can flow through two domains, either through the soil matrix or as preferential flow. Whilst in an unstructured soil (i.e., homogeneous soil matrix) water flow is mainly a function of the saturated hydraulic conductivity of the soil matrix and is primarily driven by soil texture, in structured and rooted soils, preferential flow paths may exist along rhizopores and root channels (i.e., biopores), which can influence saturated hydraulic conductivity. This complex interaction limits any simple prediction of water flow in structured soils based on soil fundamental characteristics (e.g., pedotransfer functions or Richards equation (Beven and Germann 2013)). For instance, Watson and Luxmoore (1986), who measured infiltration in a forest soil, found that 95% of water flow occurred in macropores (> 250 μm diameter), bypassing most of the soil matrix despite these macropores representing only 0.3% of the total pore volume in soil. Therefore, quantifying the changes in soil hydrological properties induced by root inclusions, and the variability associated with species and vegetation dynamics (e.g., plant life cycles), are essential. Indeed, understanding these structural and hydrological effects remain fundamental for multiple applications, including soil conservation in sustainable agriculture (Hallett et al. 2022; Marin et al. 2022), bio-tillage (Zhang and Peng 2021), slope stabilisation in ecological engineering (Sidle and Bogaard 2016) and sustainable drainage systems (Boldrin et al. 2022).

Studies on soil water infiltration and hydraulic conductivity in relation to different vegetation has shown increased soil ability to infiltrate water downwards in woodlands (Archer et al. 2015; Cheng et al. 2011). For instance, both Archer et al. (2015) and Cheng et al. (2011), working respectively in United Kingdom and China, reported greater water infiltration in woodlands compared with grasslands. Whilst most studies on woody species report an increase in hydraulic conductivity induced by plants (Ghestem et al. 2011; Greenwood and Buttle 2014; Vergani and Graf 2016), similar literature on herbaceous species presents contrasting conclusions (i.e., reporting both increases and decreases in hydraulic conductivity) (Gish and Jury 1983; Jotisankasa and Sirirattanachat 2017; Macleod et al. 2013). Boldrin et al. (2022), testing soils vegetated with six herbaceous species from three contrasting plant functional types (forbs; grasses; Legumes) and two environments (dry and wet meadows), found notable species and functional-type effects on soil hydraulic conductivity. These differences were explained by the negative relationship between specific root length and induced changes in *K*_*s*_ (Boldrin et al. 2022). The influence of roots on water flow in soil has also been shown to be highly dynamic, changing as plants grow. For instance, Leung et al. (2018) found a linear and positive relation between plant age and infiltration in soil columns vegetated with both grass (*Lolium perenne* x *Festuca pratensis*) and woody (*Salix viminalis*) species. This relationship has been explained with root development down the soil column and root turnover (i.e., development of root channels) with plant ageing.

Although literature has demonstrated the root influence on water flow in soil (Lu et al. 2020), with a limited number of studies investigating driving factors such as species and plant age (Boldrin et al. 2022; Leung et al. 2018; Vergani and Graf 2016), several open questions on a highly dynamic system remain. For example, despite knowing that root length density generally decreases exponentially down the soil profile, with herbaceous species having 44% of root biomass in the top 10 cm (Jackson et al. 1996), the relation between root-induced *K*_*s*_ changes and soil depth has not been investigated. Furthermore, although dynamic changes of soil hydrological processes have been associated with root channels after root decomposition, there remains limited information on *K*_*s*_ of rooted soils before (living roots) and after the decomposition (root channels) of contrasting root systems (Pagès 2011).

Root channels may represent preferential flow paths bypassing most of soil matrix, and so further understanding of the water redistribution from root channels (preferential flow domain) to the bulk soil (matrix domain) is needed. The properties of soil surrounding the root channels (i.e., walls of root channels) can represent boundary conditions regulating the water movement between these two domains, as well as the stability of macropores. However, data on soil physical properties within the root channels is currently severely lacking.

To fill these research gaps, we selected contrasting species from three different functional types to study the factors influencing water flow in rooted soils. These include the effects of contrasting species, soil depth, decomposition and physical properties of soil surrounding the root channels (after root decomposition).

We hypothesise that (i) *K*_*s*_ down the soil profile differs significantly between tested species and control soil; (ii) root decomposition results in a notable increase in *K*_*s*_ down the soil profile; (iii) soil surrounding the root channels has distinct physical properties compared to unrooted bulk soils. The experiments reported in this study tested these hypotheses using soil columns rooted by contrasting herbaceous species.

## Materials and Methods

### Soil

The soil used in this study was an agricultural topsoil collected from Bullionfield at The James Hutton Institute (Dundee, UK). This soil can be classified as silt loam, with 43.3% sand, 55.2% silt and 1.5% clay (BS1377). Organic matter content was 4.6% by mass (Loss on Ignition at 450° C). After collection, the soil was air-dried for a week and sieved at 2 mm.

### Plant species

Contrasting herbaceous species belonging to three different functional types (forbs, grasses, and legumes) were selected for testing in this study (Table 1). The choice of species was based on contrasting results in a previous study on plant influence on soil physical properties (Boldrin et al. 2022), as well as differing root traits (e.g., fibrous roots vs taproots). Details on the original species selection are provided in Boldrin et al. (2022).

**Table 1:** A list of the species selected for testing in this study. Their family, functional type, common name and the acronym used throughout this study are reported

Species	Family	Functional type	Com. name	acronym
*Daucus carota*	Apiacea	Forb	Wild Carrot	F-DC
*Deschampsia cespitosa*	Poacea	Grass	Tufted Hair-grass	G-DC
*Festuca ovina*	Poacea	Grass	Sheep’s Fescue	G-FO
*Lotus corniculatus*	Fabacea	Legume	Birdsfoot Trefoil	L-LC

### Saturated hydraulic conductivity in large soil columns

Soil columns (height = 0.45 m = 0.40 m soil + 0.05 m pea-gravel drainage-layer) comprised of PVC drainage pipes (100 mm inner diameter and 0.5 m long) vegetated with three plants of the same species (pre-germinated seeds). All soil columns were randomly arranged in an unheated glasshouse with no additional light or heat provided. The glasshouse temperature and relative humidity were therefore close to the outdoor conditions at The James Hutton Institute (Dundee, UK). After plants were allowed to grow for a period of 3 months (April – June; temperature and humidity close to outdoor conditions), hydraulic conductivity (*K*_*s*_) was measured by applying a constant ponding head (30 mm) with a Mariotte bottle, whilst allowing free drainage at the column base. After testing, soil columns were removed from pipes and sectioned into three cores of 13 cm. Roots in each section were washed from the soil in gently running tap water on a set of sieves with a range of sieve sizes, from 2.0 to 0.5 mm mesh. Roots at the three soil depth ranges were imaged on a flatbed scanner and analysed using WinRhizo software (Regent Instruments Inc.) to determine total root length. Following length determination, all roots were oven-dried at 70° C to measure root biomass. Root density (root-mass per soil volume) and root length density (root length per soil volume) were determined in the three sections (i.e., layers) per each column.

### Hydraulic conductivity down vegetated soil

**Fig. 1:**
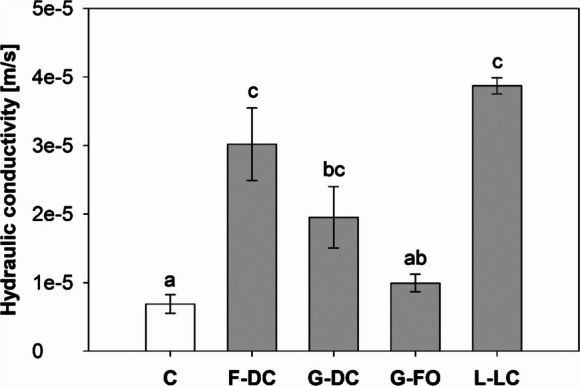
Saturated hydraulic conductivity of control (C, fallow soil) and vegetated soil columns with contrasting species. Means are reported ± standard error of mean (*n* = 5). Different letters the graph indicate a statistically significant difference between treatments, as tested using one-way ANOVA (*F-value* = 14.37; *p-value* < 0.001) followed by post hoc Tukey’s test. Acronyms: C (Control); F-DC (*Daucus carota*); G-DC (*Deschampsia cespitosa*); G-FO (*Festuca ovina*); L-LC (*Lotus corniculatus*)

Following initial testing of *K*_*s*_ in large soil columns, a second experiment was established to determine the change in *K*_*s*_ caused by contrasting species down the soil profile. Seeds were pre-germinated on filter paper in Petri dishes (20.00 °C in the lab) and individual plants of the four contrasting species (four replicates per species) were grown in soil columns contained in transparent-acrylic tubes (50 mm internal diameter; 315 mm height; see Suppl.Fig. 1) with the bottom covered by a 0.2-mm polyester mesh to allow water drainage. Each acrylic cylinder was comprised of five individual sections (50 mm diameter and 60 mm length) connected by electrical tape and covered with reflective sheet to maintain roots in dark conditions. On the top of the first 60-mm section, an extra 20 mm ring was added to facilitate irrigation and soil surface removal (3 mm) before testing. The first 3 mm of soil (i.e., surface) was excluded to avoid any effect induced by algal growth on the soil surface. At the interface between acrylic-pipe sections, 3 mm acrylic spacers (small pipe sections) were placed to favour the slicing of the soil column in the five sections (i.e., five cores in the acrylic-pipe rings). Soil in the columns was packed to a target dry bulk density of 1.35 g/cm^3^ at optimum water content of 0.18 g/g. Prior to transplanting, all soil columns were saturated and left to drain for 24 h (i.e., reaching field capacity) to provide an optimum environment for the establishment of the pre-germinated seeds. During plant establishment (July – October), all soil columns were subjected to the same irrigation schedule ranging from 10 to 15 ml, two times per week, to avoid any form of plant water stress or soil surface drying and shrinking (i.e., water was supplied ad libitum). All soil columns were randomly arranged in an unheated glasshouse with no additional light or heat provided. The glasshouse temperature and relative humidity were thus close to the outdoor conditions at The James Hutton Institute (Dundee, Scotland). Control fallow soil columns were subjected to the same glasshouse conditions and irrigation regime.

Following four months of plant growth, the columns were split into five sections (60 mm each) and *K*_*s*_ was tested in each section (i.e., down soil depth) using a constant-head permeameter (5-station Chameleon kit, Soilmoisture, USA). Control columns (C) were tested identically.

### Hydraulic conductivity after root decay

After initial plant growth and *K*_*s*_ testing for each cylinder section (i.e., soil cores down each column), sample cores (rooted and control soils) were randomly divided in two groups (two replicate columns per group). In the first group, the cores, tested for *K*_*s*_, were placed in boxes with a gravel-drainage layer and buried using the same soil used in the cores (silt loam). Soil water content was maintained close to field capacity by scheduled irrigation and all the boxes were maintained in a heated glasshouse (18 °C (night) – 25 °C (day)) for seven months. The second group of cores was stored in a cold store at 5 °C for seven months. The two contrasting treatments aimed to mimic conditions favouring (e.g., high temperature and moisture) or slowing down (e.g., low temperature) root decomposition in soil, as well as conditions that may occur during sample storage (e.g., sampled cores in cold store). After seven-months incubation under the contrasting conditions, all soil cores were re-tested for *K*_*s*_. Cores vegetated with *F. ovina* were not subjected to the decomposition treatment because *K*_*s*_ testing showed no differences between the control and *F. ovina* vegetated-soils.

In a separate linked experiment, a second set of soil columns (4 reps.; 8 columns) was tested after plant growth (4 months) and following root decay (7-months buried in soil in a heated glasshouse) for the most contrasting treatments (fallow soil and *D. carota* vegetated-soils). This was to provide independent replicate of the experiment on *K*_*s*_ alteration down the soil-profile by root decomposition.

All tested soil-cores (i.e., column sections) were oven-dried (105 °C) at the end of the experiment and dry bulk density was determined.

A subset of representative cores (i.e., different soil columns and treatments) was imaged after root decomposition in X-ray micro-CT scanner (Nikon XT H 225ST micro-CT scanner) using a reflection target with tungsten metal, 1 mm copper filter and 1000 ms exposure time. The detector of the micro-CT scanner was a Perkin Elmer 1620, carbon fibre structured detector, with 2000 × 2000-pixel resolution. During each CT-scan, 3142 images were taken (360° full turn). These images were reconstructed using VGStudio Max version 3.4 to obtain a 3D volumetric representation of each scanned core. Micro-CT images provided visual context on the development of root channels down the soil columns. It was not in the scope of this study to extract quantitative information from the CT images.

### Longitudinally divided soil-columns for root-channel testing

**Fig. 2:**
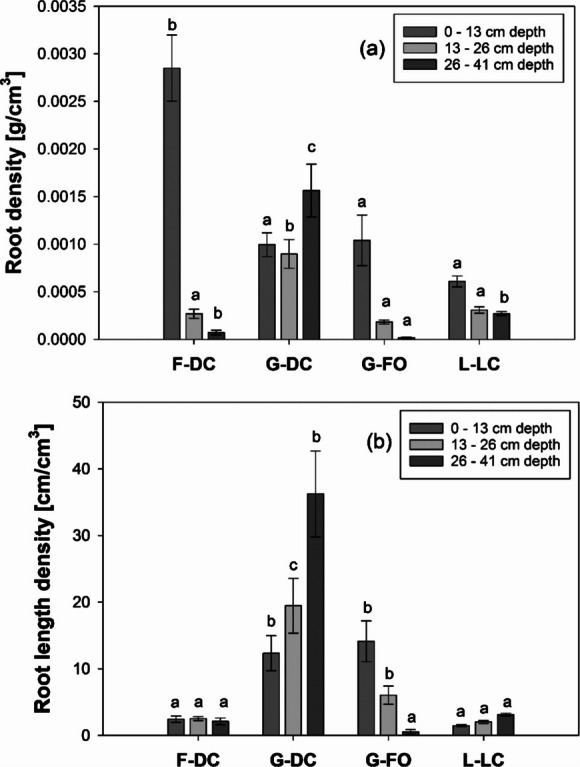
(**a**) Root density (root biomass per soil volume) and (**b**) root length density (root length per soil volume) down the soil profile (three depth ranges in soil columns). Three plants per each soil column (diameter = 100 mm; plants/m.^2^ = 382). Means are reported ± standard error of mean (*n* = 5). Acronyms: F-DC (*Daucus carota*); G-DC (*Deschampsia cespitosa*); G-FO (*Festuca ovina*); L-LC (*Lotus corniculatus*). Letters indicate a statistically significant difference between species at the same soil depth, as tested using one-way ANOVA followed by post hoc Tukey’s test. [Root density (i) 0–13 cm: *F-value* = 19.15, *p-value* < 0.001; (ii) 13–26 cm: *F-value* = 15.97, *p-value* < 0.001; (iii) 13–26 cm: *F-value* = 30.70, *p-value* < 0.001 (log-transformed); Root length density (i) 0–13 cm: *F-value* = 30.89, *p-value* < 0.001 (log-transformed); (ii) 13–26 cm: *F-value* = 39.01, *p-value* < 0.001 (log-transformed); (iii) 26–41 cm: *F-value* = 59.87, *p-value* < 0.001 (Square root transformed)]

Individual plants (pre-germinated seeds) of *D. carota; D. cespitosa and L. corniculatus* were grown in the top-half of a soil column (300 mm height; 50 mm width; five-replications per treatment; Suppl.Fig. 2) longitudinally divided by a 40-μm nylon-mesh, that cannot be penetrated by roots (McKenzie et al. 2009). The soil in the columns was packed in ten layers with a dry bulk density of 1.2 g/cm^3^ at a water content of 0.18 g/g. During packing, the surface of each soil layer was abraded to achieve a better contact between each successive layer. Before transplanting pre-germinated seeds of the contrasting species, all the columns were fully saturated with water and left to drain for 24 h to provide appropriate moisture for the germinated seeds (i.e., field capacity). During plant establishment (five-months), columns were randomly arranged in an unheated glasshouse and inclined at a 15-degree angle to the vertical to favour root growth at the soil-mesh interface. All columns were equally irrigated with 10–15 ml of water twice a week. Five fallow soil columns (control) were subjected to the same conditions as the vegetated soil columns.

Following plant establishment, plants were killed by a single glyphosate-herbicide application and left for root decomposition. During the decomposition period, all soil columns (rooted and control fallow) were buried in boxes filled with silt loam soil above a gravel-drainage layer. The boxes with the buried soil columns were placed in a heated glasshouse (18–25 °C) for seven-months to favour decomposition. Soil moisture in the boxes was maintained close to field capacity. After the decomposition period, soil columns were split along the centreline, and the mesh was removed to expose the root channels following the decay of roots.

### Physical properties of soil sounding the root channels

After splitting the soil columns, the top-half of each soil column (i.e., containing the root channels) was placed on a ceramic suction plate (Soilmoisture, USA) to establish moisture equilibrium at a suction of 10 kPa. Soil surrounding the root channels was then tested for hydro-mechanical properties at two depths down each column: 50–100 mm and 200–250 mm. Within the control soil columns (fallow soil) tests were performed in the bulk soil.

Soil mechanical properties were quantified by indentation testing using a universal testing frame (Instron 5966, Norwood, MA, USA) fitted with a 50 N load cell and a 3-mm-diameter spherical indenter. In each soil column, indentation tests were carried out on root-channel soil at both 50–100 mm and 200–250 mm depths to account for depth influence on mechanical properties. During each test, the indenter penetrated at a rate of 1 mm min^−1^ to 1 mm depth (loading) before being removed at the same rate (unloading). Further details of the indentation method and calculations for determining hardness and elasticity are provided in Naveed et al. (2018).

At the same depth of indentation tests, water sorptivity and hydrophobicity were tested using a miniaturised infiltrometer developed by Hallett et al. (2003) to test the hydraulic properties of the rhizosphere. Water sorptivity describes the soil’s ability to absorb and transmit water. Ethanol sorptivity was also tested due to the non-polar nature of ethanol (non-influenced by hydrophobicity). During tests, sorptivity was measured using a 0.4 mm diameter glass capillary tube connected by tubing to a reservoir, with water or ethanol, on an analytical balance (resolution = 0.0001 g). Infiltration tests were performed at −5 mm hydraulic head. The rate of liquid uptake from the reservoir by the root-channel soils was recorded by the balance as reduction in mass at 1-s intervals for 100 s. The slope of the linear relation between mass reduction (i.e., liquid absorption) and time represents the mass flow rate (*Q*) of infiltration and can be used to calculate sorptivity (*S*) (Eq. 1):

1 $$S=\sqrt{\frac{Qf}{4br}}$$

where the *f* is the fillable air porosity of the soil; *b* depends on the soil–water diffusivity function with an average value equal to 0.55 (varying between 0.5 and 0.78); *r* is the radius of the 0.4-mm glass-infiltrometer tip (Naveed et al. 2018).

Hydrophobicity was estimated comparing water and ethanol sorptivity. Hydrophobicity index (*H*_*yd*_) was calculated as

2 $${H}_{yd}=1.95\frac{{S}_{E}}{{S}_{W}}$$

where *S*_*E*_ and *S*_*W*_ are the sorptivity of ethanol and water, respectively. The multiplier 1.95 accounts for the differences in surface tension and viscosity of the two liquids. A greater value of the index indicates a more hydrophobic soil.

Small soil cores (19 mm internal diameter; 11 mm height) were sampled down the soil profile from root channels (i.e., along the exposed soil in the column) at 50–100, 150 (middle depth) and 200–250 mm depth. The cores collected at 50–100 mm and 200–250 mm depths were tested for soil water retention curves (SWRCs). Drying SWRCs were measured using a ceramic suction plate (for matric suction from 5 to 50 kPa) and a pressure plate apparatus (300 and 1500 kPa pressure; Soilmoisture Equipment Corp, USA). Water content was expressed as gravimetric water content (i.e., water mass per soil dry mass) as this was deemed more robust than volumetric water content when dealing with small cores with irregular surfaces profile (i.e., making volume-determination difficult). The measured SWRCs were then fitted using the Van Genuchten (1980) model:

3 $$w={w}_{r}+\frac{{w}_{s}-{w}_{r}}{{\left[{1+\left|\alpha \Psi \right|}^{n}\right]}^{m}}$$

where *w* is the soil water content (g/g), *w*_*r*_ is the residual soil water content at 1500 kPa (g/g), *w*_*s*_ is the saturated soil water content (g/g), *Ψ* is the soil matric suction (kPa); and *α*, *n*, and m are parameters dependent upon the shape of the curve, *m* = 1 − 1/*n*, 0 < *m* < 1.

Soil collected from cores sampled from the middle of the columns (150 mm depth) were air-dried and then tested for stability in water using a wet sieving apparatus (Eijelkamp Soil & Water, Netherlands). In the wet sieving apparatus, the air-dried cores (small soil cylinders) were placed on 2-mm-mesh sieves, and then mechanically moved up and downward (up-down strokes = 1.3 cm) in a can filled with distilled water for 3 min ± 5 s (34 times/min up-down strokes). Unstable soil fell apart and passed through the 2 mm sieve into the water-filled can placed under the sieve. Subsequently, the cans with soil from unstable aggregates were removed and replaced by new water-filled cans. All stable soil on the sieve mesh was fully pulverised and soil from the stable aggregates was collected into the water can. The soil had no particles greater than 2 mm (i.e., sieved before column-packing). After oven-drying the two sets of cans with the unstable and stable soils, soil stability in water (*S*_*SW*_) was calculated using the weight of stable (*S*_*S*_) and unstable (*S*_*U*_) soil:

4 $${S}_{Sw}=\frac{{S}_{S}}{\left({S}_{S}+{S}_{U}\right)}$$

### Statistical analysis

Statistical analysis was performed using GenStat 17th Edition (VSN International) and SigmaPlot13 (Systat Software Inc). Significant differences were assessed with one way-ANOVA, followed by post-hoc Tukey's test. Non-normal data were log or square root transformed before ANOVA tests. Results were considered statistically significant when *p-value* ≤ 0.05. The variability in the averaged result is presented as ± standard error of mean. Details (e.g., *F-values*, *p-values*, data-transformation) of statistical analyses for each dataset are provided in figure captions and in Supplementary Table 1.

## Results

On average, vegetated soils showed 3.5-times greater *K*_*s*_ compared with the control fallow soils (6.88e-6 ± 1.37e-6 m/s). However, large differences between species existed (Fig. 1). Whilst *K*_*s*_ in columns vegetated with the grass *F. ovina* did not significantly differ from that of the control soils, the columns vegetated with the legume *L. corniculatus* (3.87e-5 ± 1.17e-6 m/s) showed 5.6-times greater *Ks* than the control soils. The grass *D. cespitosa* had intermediate values of *K*_*s*_ (1.95e-5 ± 4.49e-6 m/s).

Root density and root length density varied significantly between the tested species (Fig. 2). Root density (biomass per soil volume) showed a sharp decrease with soil depth. While the tap-root system of *D. carota* exhibited large root density (a) and small root length density (b) values in the top-soil, *D. cespitosa* and *F. ovina* (fibrous roots) generally showed smaller biomass and greater length in the same soil layer.

**Fig. 3:**
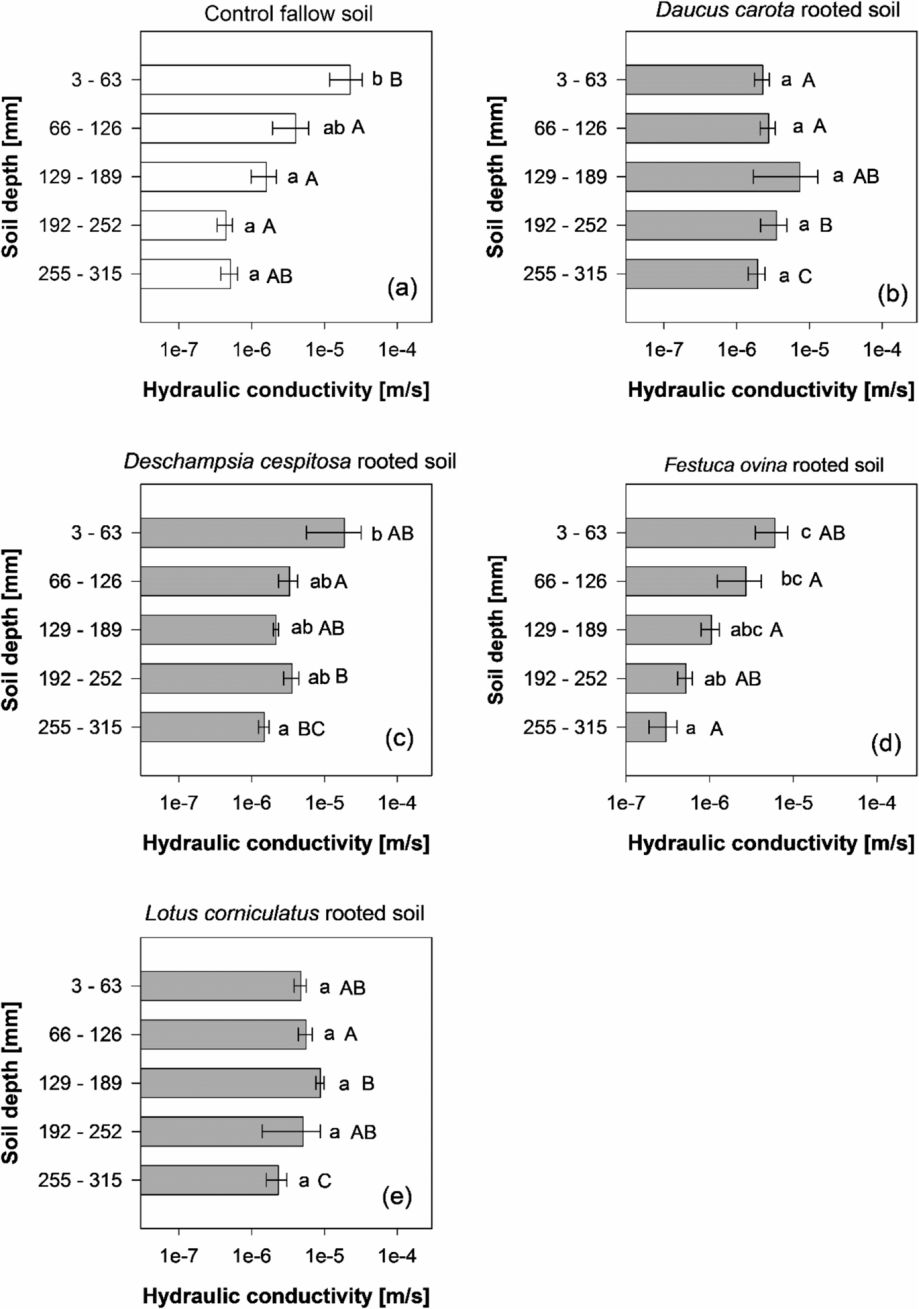
Saturated hydraulic conductivity down the soil profile in (**a**) control fallow soil and (**b**) *Daucus carota* vegetated soil; (**c**) *Deschampsia cespitosa* vegetated soil; (**d**) *Festuca ovina* vegetated soil and (**e**) *Lotus corniculatus* vegetated soil. Means are reported ± standard error of mean (*n* = 4). Different letters in the graphs indicate a statistically significant difference between treatments, as tested using one-way ANOVA followed by post hoc Tukey’s test. Lowercase letters indicate statistical differences between layers (i.e., down the soil profile) in the same treatment (e.g., species). Capital letters indicate statistical differences between treatments at the same soil depth [detailed results of statistical analysis are given in Suppl. Table 1]

The influence of roots on soil properties is generally a function of root depth with large differences between species. Therefore, the initial observation of an increase in *K*_*s*_ might be associated with both larger values in shallow soil layers, as well as a *K*_*s*_ increase down the soil profile. Results in Fig. 3 reveal that the plant effects on *K*_*s*_ are not limited to the soil surface (i.e., at the root-shoot transition zone or shallow soil layer); notable differences exist between species regarding their ability to modify *K*_*s*_ down the soil columns. In control soil, a sharp and exponential decrease in *K*_*s*_ with increasing depth was observed. Whilst the first layer of the control column (3–63 mm depth) showed large *K*_*s*_ values (2.26e-5 ± 1.08e-5 m/s), the *Ks* values obtained from the third (129–189 mm depth) and fourth (192–252 mm depth) layers exhibited 93% (1.59–6 ± 6.05e-7 m/s) and 98% (4.42e-7 ± 1.05e-7 m/s) drops, respectively, compared to the first layer (Fig. 3a). In contrast, columns vegetated with *D. carota* and *L. corniculatus* showed smaller *K*_*s*_ values in shallow soil layers, compared to the control (F-DC = 2.29e-6 ± 5.29e-7; L-LC = 4.73e-6 ± 8.66e-7; Fig. 3b,e)), and also a consistent increase in *K*_*s*_ from 3–63 mm to 129–189 mm depth (F-DC = + 222%; L-LC = + 87%). Therefore, in soils vegetated with *D. carota* and *L. corniculatus*, *K*_*s*_ peaked at 129–189 mm depth (Fig. 3b, e). Columns vegetated with grass species exhibited *K*_*s*_-depth trends similar to the control columns, and maximum *K*_*s*_ values similarly at 3–63 mm depth (Fig. 3c,d). The columns vegetated with the grass *F. ovina* showed no *K*_*s*_-depth differences with respect to the control columns (Fig. 3d)).

**Fig. 4:**
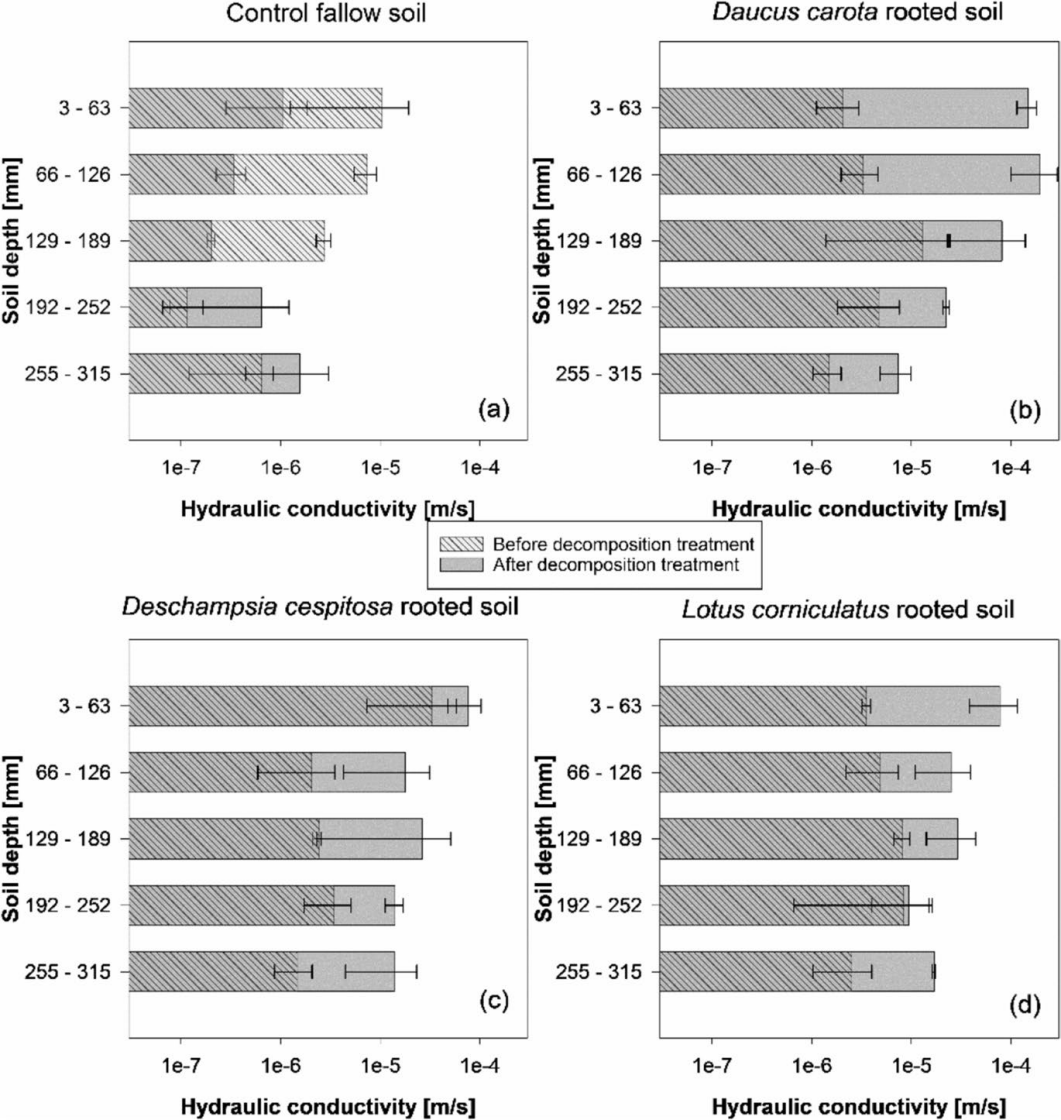
Saturated hydraulic conductivity down the soil profile before (area with oblique-lines pattern) and after the decomposition period in the heated glasshouse for 7-months (grey area) in (**a**) control fallow soil and (**b**) *Daucus carota* vegetated soil; (**c**) *Deschampsia cespitosa* vegetated soil; (**d**) *Lotus corniculatus* vegetated soil. Means are reported ± standard error of mean (*n* = 2)

Following the seven-month decomposition treatment in the heated-glasshouse, all rooted cores showed a notable increase in *K*_*s*_ (Fig. 4b,c,d). In contrast, unvegetated soil-cores (control columns) showed a decrease in *K*_*s*_ compared to the measurements made before the decomposition treatment (Fig. 4a). The greatest *K*_*s*_ increases after treatment were observed in the 3–63 mm-depth cores from *D. carota* (82-times increase) and *L. corniculatus* (21-times increase) columns. Although all cores from vegetated columns exhibited a *K*_*s*_ increase following the decomposition treatment, a reducing trend with depth was observed (Fig. 4b,c,d). Indeed, maximum *K*_*s*_ values in *D. carota* and *L. corniculatus* were recorded in the topsoil layers. Therefore, the entire *K*_*s*_-depth relationships in the vegetated columns were altered by the decomposition treatment in the heated-glasshouse. Interestingly, the grass *D. cespitosa* showed greater *K*_*s*_ increases in the 129–189 mm depth core compared to the shallow soil layer (3–63 mm) and hence an opposite trend compared to other species (Fig. 4c).

**Fig. 5:**
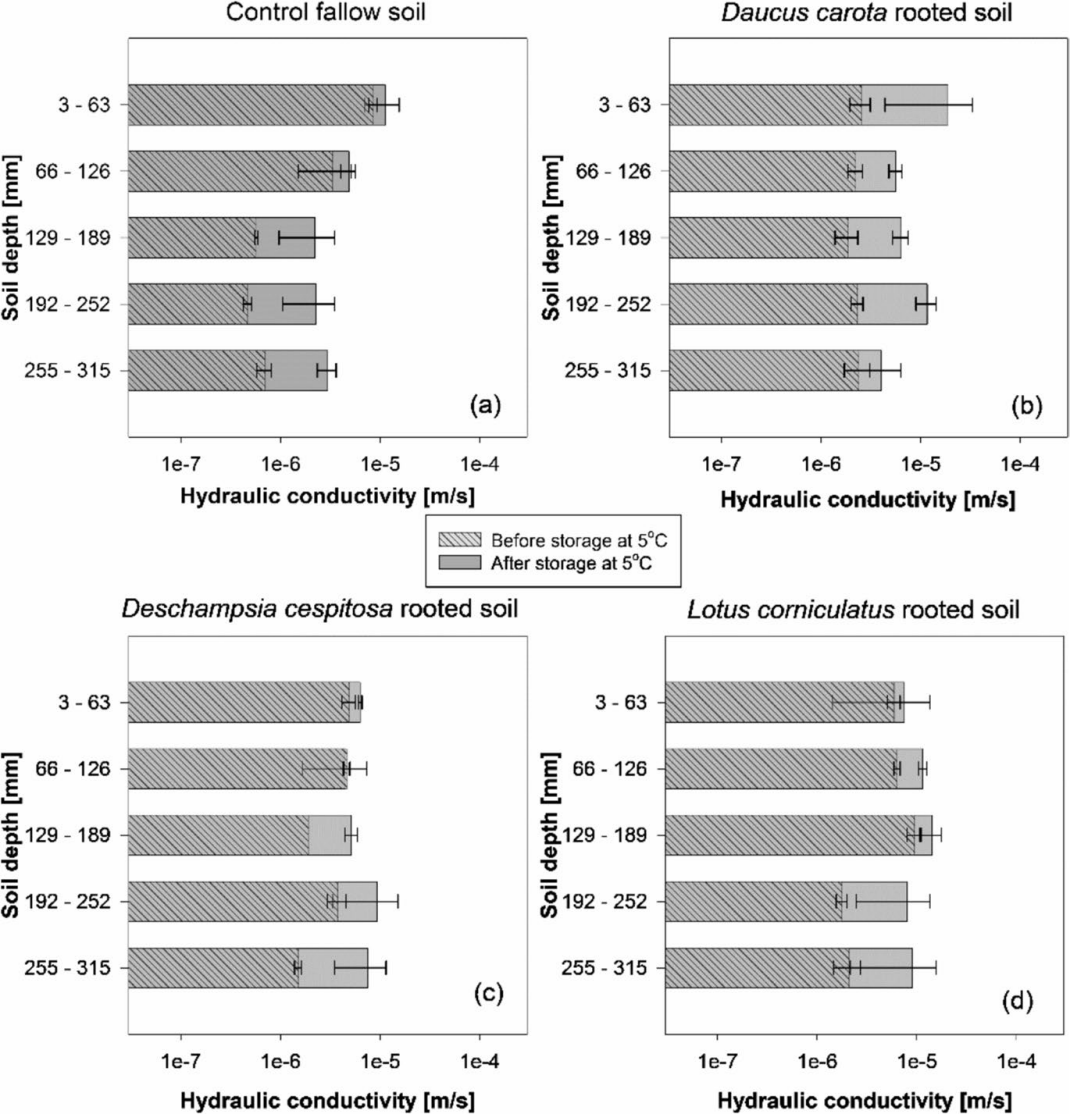
Saturated hydraulic conductivity down the soil profile before (area with oblique-lines pattern) and after the period (7-months) in a cold store at 5 °C (grey area) in (**a**) control fallow soil and (**b**) *Daucus carota* vegetated soil; (**c**) *Deschampsia cespitosa* vegetated soil; (**d**) *Lotus corniculatus* vegetated soil. Means are reported ±standard error of mean (*n* = 2)

After seven-months in a cold store at 5° C, *K*_*s*_ showed a general increase. However, the magnitude of change was lower when compared to the observed *K*_*s*_ increases after the decomposition-treatment in the heated-glasshouse. For instance, whilst after the decomposition-treatment in the heated-glasshouse, *D. carota* and *L. corniculatus* cores at 3–63 mm depth exhibited respectively 82- and 21-times increases in *K*_*s*_, after the same period in the cold store, *K*_*s*_ increases for *D. carota* and *L. corniculatus* cores at the same depth were respectively 10- and 2-times (Figs. 4b,d and 5b,d). No clear trend of *K*_*s*_ change with depth was observed in the samples kept at 5° C (Fig.5).

Dry bulk density, measured after *K*_*s*_ testing, showed a progressive increase with soil depth in all treatments (Table 2). For instance, dry bulk density in the control columns ranged between 1.31 ± 0.01 g/cm^3^ in the top layer to 1.37 ± 0.01 g/cm^3^ in the bottom layer.

**Table 2:** Dry bulk densities [g/cm^3^] in the five tested layers (i.e., cores) down the soil columns per each treatment. Acronyms: fallow control columns [C], *Daucus carota* [F-DC]; *Deschampsia cespitosa* [G-DC], *Lotus corniculatus* [L-LC]

Depth, mm	*C—*bulk density [g/cm^3^]	*F-DC-* bulk density [g/cm^3^]	*G-DC-* bulk density [g/cm^3^]	*L-LC-* bulk density [g/cm^3^]
3—63	1.31 ± 0.01	1.36 ± 0.01	1.30 ± 0.02	1.33 ± 0.01
66—126	1.32 ± 0.01	1.36 ± 0.01	1.37 ± 0.03	1.36 ± 0.01
129—189	1.35 ± 0.02	1.33 ± 0.01	1.36 ± 0.01	1.31 ± 0.03
192—252	1.37 ± 0.01	1.33 ± 0.01	1.35 ± 0.01	1.38 ± 0.01
255—315	1.37 ± 0.02	1.41 ± 0.01	1.38 ± 0.01	1.38 ± 0.01

**Fig. 6:**
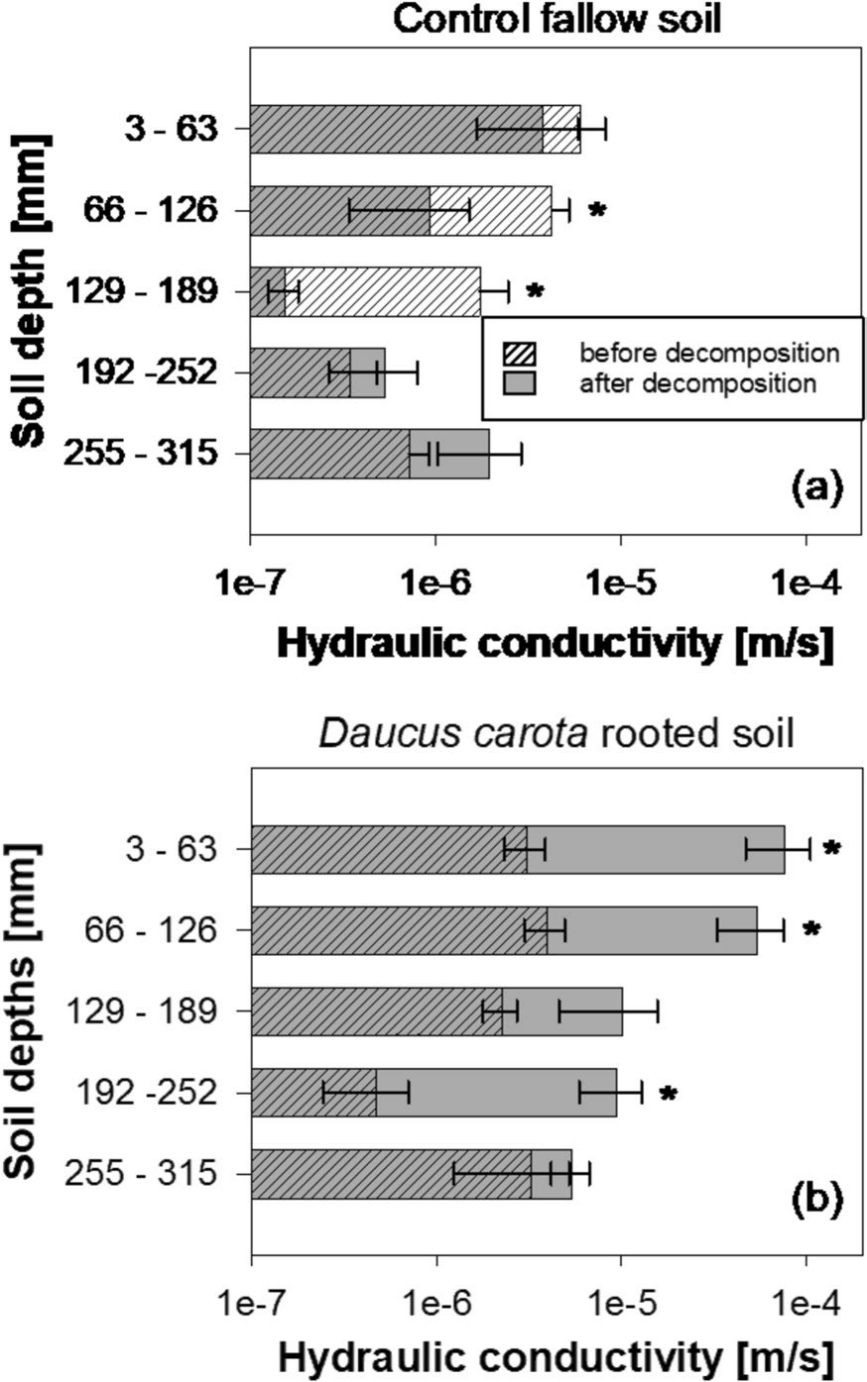
Saturated hydraulic conductivity down the soil profile before (area with oblique-lines pattern) and after (grey area) the decomposition period in (**a**) control fallow soil and (**b**) *Daucus carota* rooted soil. Means are reported ± standard error of mean (*n* = 4). * indicates a statistically significant difference between before and after decomposition-treatment tested by one-way ANOVA (log-transformed data) [(**a**) Control soil: 66–126 mm: *F-value* = 11.14, *p-value* = 0.016; 129–189 mm: *F-value* = 6.06, *p-value* = 0.049; (**b**) *D. carota* soil: 3–63 mm: *F-value* = 37.70, *p-value* < 0.001; 66–126 mm: *F-value* = 8.24, *p-value* = 0.028; 192–252 mm: *F-value* = 14.29, *p-value* = 0.009)

**Fig. 7:**
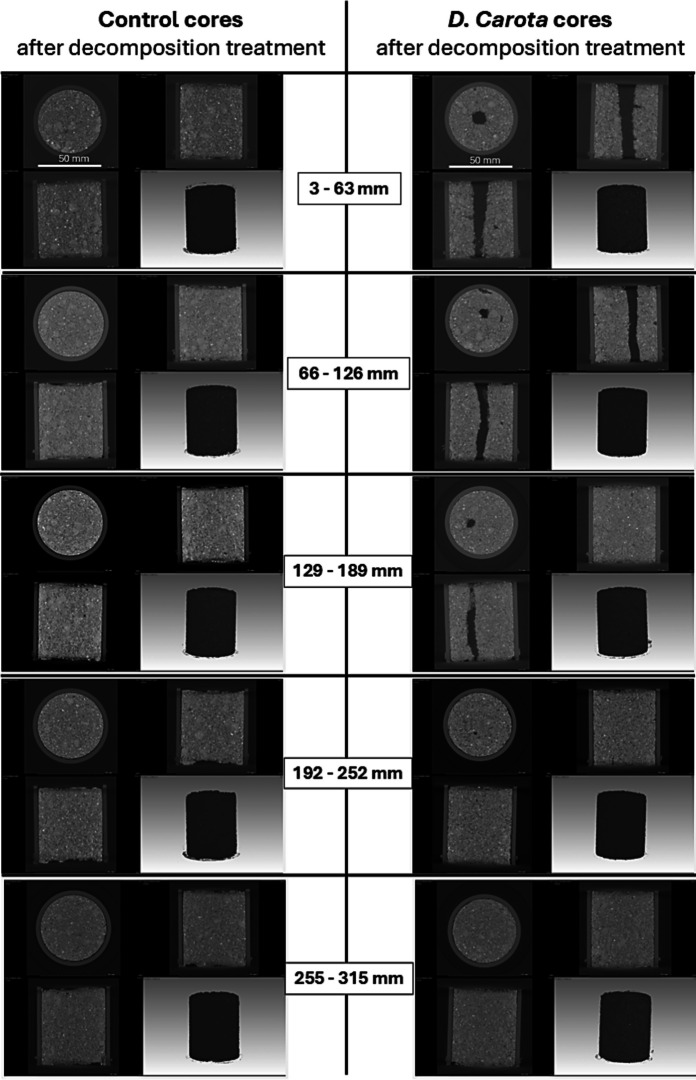
Example images of control and *D. carota* cores from two soil columns obtained with X-Ray micro CT scanner. For each core (i.e., depth): top-left picture = top-view; top-right picture = side view; bottom-left picture = side view after 90° rotation; bottom-right picture = 3D image of core. Each core has 50 mm diameter of 60 mm height

A second set of soil columns was tested to validate the abrupt increase in *K*_*s*_ in columns vegetated with *D. carota* after the decomposition-treatment. This second set of tests was able to confirm the previous data and enhance robustness of results on *K*_*s*_ changes with root decomposition (Fig. 6). Indeed, similarly to previous results (only two replicate columns), these tests (four replicate columns) highlighted an abrupt increase in *K*_*s*_ after the decomposition-treatment when soil was vegetated with *D. carota* (Fig. 6). In contrast, control unvegetated soil showed a decrease in *K*_*s*_ when retested (i.e., after seven-months decomposition treatment) (Fig. 6a). Figure 6a and b show the relationships between *K*_*s*_ and depth before and after the decomposition treatment in the control and the *D. carota* columns. Large continuous root-channels were observed from the X-ray micro-CT images taken from these columns (Fig. 7).

**Fig. 8:**
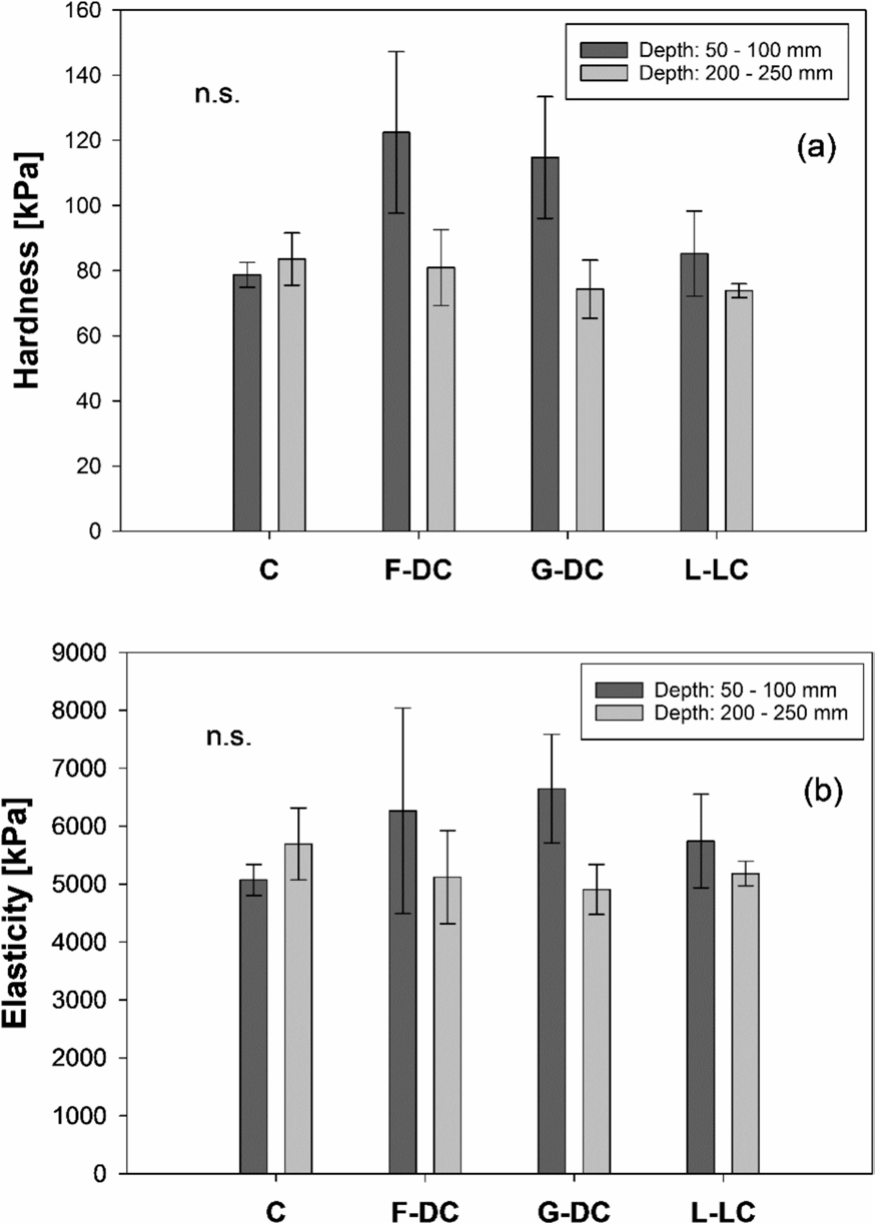
(**a**) Hardness and (**b**) elasticity measured in the bulk soil of unvegetated columns [C] and in the root channels created by the root decomposition of contrasting species in the vegetated columns (*Daucus carota* [F-DC], *Deschampsia cespitosa* [G-DC], *Lotus corniculatus* [L-LC]) at 50–100 and 200–250 mm depth down the profile of longitudinally-divided column. Means are reported ± standard error of mean (*n* = 5). *n.s.* indicates the lack of statistically significant differences

Although the results depicted in Figs. 1 and 3 highlighted a bulk increase in water flow down the soil profile due to the presence of roots, and Figs. 4 and 6 indicated a further increase in *K*_*s*_ after root decomposition following the development of root channels (i.e., bio-pores) creating preferential-flow paths (Fig.7), water redistribution in the bulk-soil surrounding these channels depends on the soil properties in the root channel (i.e., channel walls). Indentation tests found a tendency to hardness and elasticity increase in *D. carota* and *D. cespitosa* root channels in shallow soil (50–100 mm) compared to the control soils (i.e., no vegetation, no root channels; Fig.8). However, the large variability resulted in no observable significant differences between treatments. In deeper soil layers (200–250 mm), where root channels were generally smaller and less marked, no differences were observed between treatments.

**Fig. 9:**
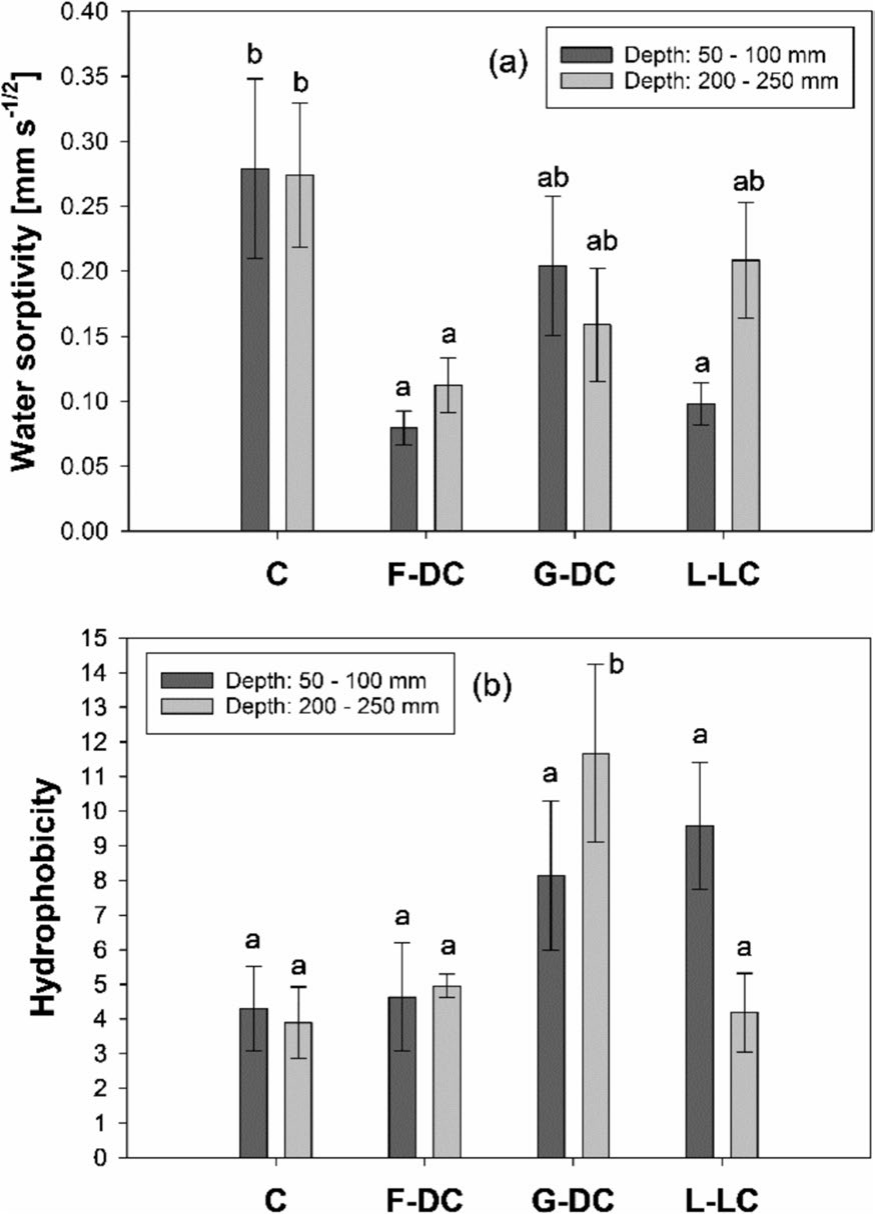
(**a**) Water sorptivity and (**b**) hydrophobicity measured in the bulk soil of fallow control columns [C] and in the root channels created by root decomposition of contrasting species (*Daucus carota* [F-DC], *Deschampsia cespitosa* [G-DC], *Lotus corniculatus* [L-LC]) at 50–100 and 200–250 mm depth down the profile of longitudinally-divided columns. Means are reported ± standard error of mean (*n* = 5). Different letters in a and b graphs indicate a statistically significant difference between treatments, as tested using one-way ANOVA followed by post hoc Tukey’s test [(**a**)Water sorptivity (log-transformed)—50–100 mm: *F-value* = 5.16, *p-value* = 0.011; 200–250 mm: *F-value* = 3.25, *p-value* = 0.049; (**b**) Hydrophobicity—50–100 mm: *F-value* = 2.27, *p-value* = 0.119; 200–250 mm: *F-value* = 6.03, *p-value* = 0.006]

Water sorptivity measurements highlighted notable differences between the control soil and root-channel soils and also differences between root channels of different species (Fig. 9a). In particular, root channels which developed after the decomposition of *D. carota* roots had a significantly reduced ability to absorb and transmit water (−72% at 50–100 mm) compared to the control soil. Similarly, a decrease in sorprivity was observed in *L. corniculatus* root-channels in shallow soil. The estimated hydrophobicity derived from the water and ethanol sorptivity showed 3-times greater values in the root channels of *D. cespitosa* at 200–250 mm depth compared with the control soil (Fig. 9b). In contrast, *D. carota* root-channels did not differ from control soil in terms of hydrophobicity.

**Fig. 10:**
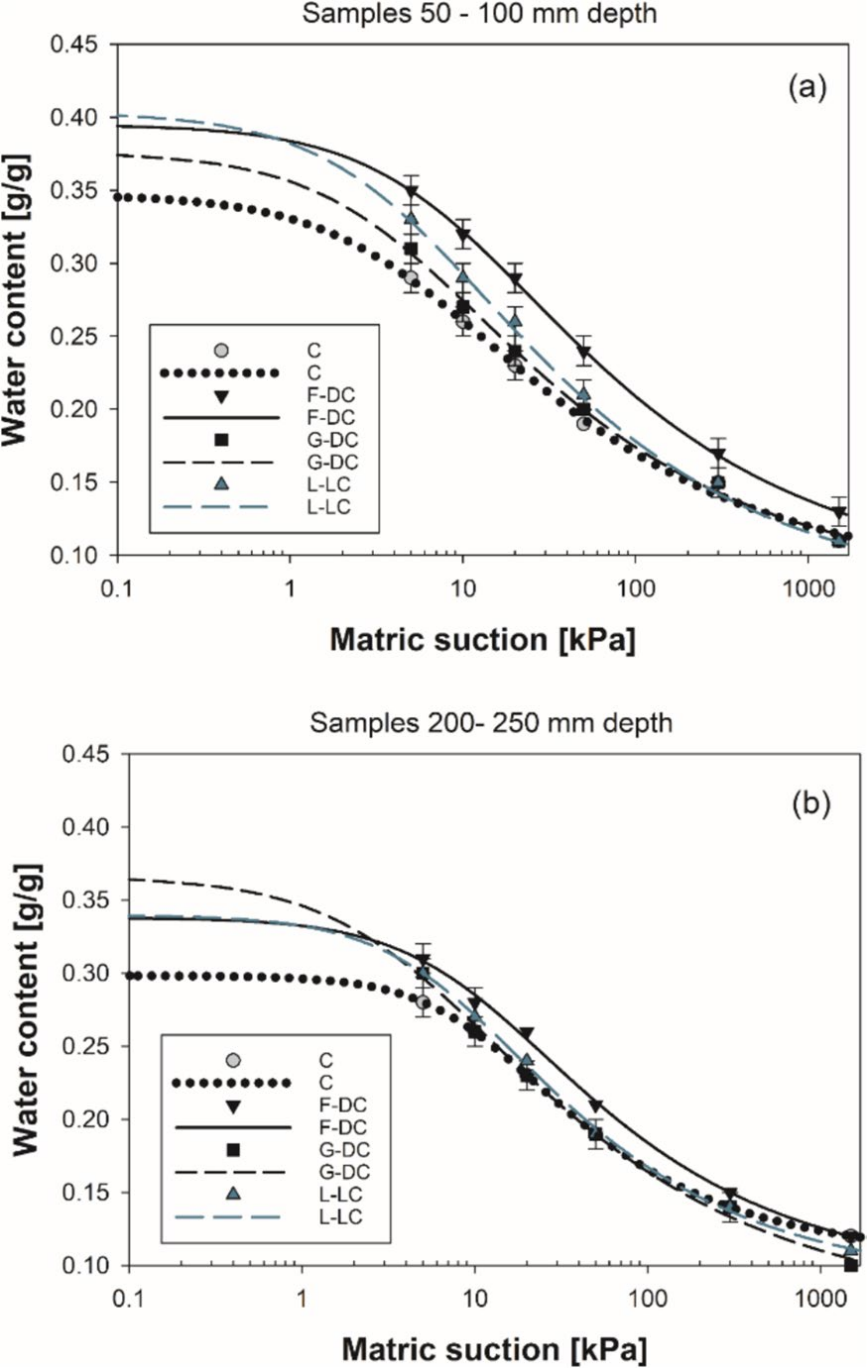
Drying soil water retention curves (SWRCs) based on gravimetric water content. SWRCs were tested in small cores sampled from each longitudinally-divided column (fallow soil and vegetated) in correspondence of root channels (vegetated soil) and in bulk soil (fallow control columns) at (**a**) 50–100 mm and (**b**) 200–250 mm depth down the soil profile. Treatments (e.g., species) are represented by different symbols. Means are reported ± standard error of mean (*n* = 5). Acronyms: fallow control columns [C]; *Daucus carota* [F-DC]; *Deschampsia cespitosa* [G-DC]; *Lotus corniculatus* [L-LC]. Data are fitted using Van Genuchten (1980) model

SWRCs of small cores sampled on root channels differed from those of the control soil, sampled at the same depths (50–100 mm and 200–250 mm) down the soil columns. Soils from the root channels had greater water content for matric suction ranging from 5 to 1500 kPa, compared to the control soil. This difference between root-channel and control soil was greater at the wet end of the SWRCs (5–20 kPa) for soils sampled between 50- and 100-mm depth (Fig.10a). Although the root-channel soils from different species generally retained more water than the control soil, notable differences between species existed, with *D. carota* root-channels having the largest effect on water retention. In contrast, the root channels developed after the decomposition of *D. cespitosa* roots had a small influence. *L. corniculatus* root-channels showed intermediate values, compared with the *D. carota* and *D. cespitosa* cases, but the effect of these channels on water retention (i.e., greater water holding capacity) quickly disappeared as soil dried to above 20 kPa suction (Fig. 10a). In the deeper soil layer (200–250 mm), treatments exhibited small differences in terms of water retention, but consistently greater values were recorded in the *D. carota* soils. Observed differences in SWRCs reflect different values of theoretically available water for plants (water content between 5 and 1500 kPa). Root-channel samples had 17% and 16% greater plant available water (PAW) in shallow (50–100 mm) and deep (200–250 mm) soil layers, respectively (at 50–100 mm: root-channels PAW = 0.21 ± 0.00 g/g, control PAW = 0.18 ± 0.01 g/g; at 200–250 mm: root-channels PAW = 0.19 ± 0.00 g/g, control PAW = 0.17 ± 0.01 g/g).

**Fig. 11:**
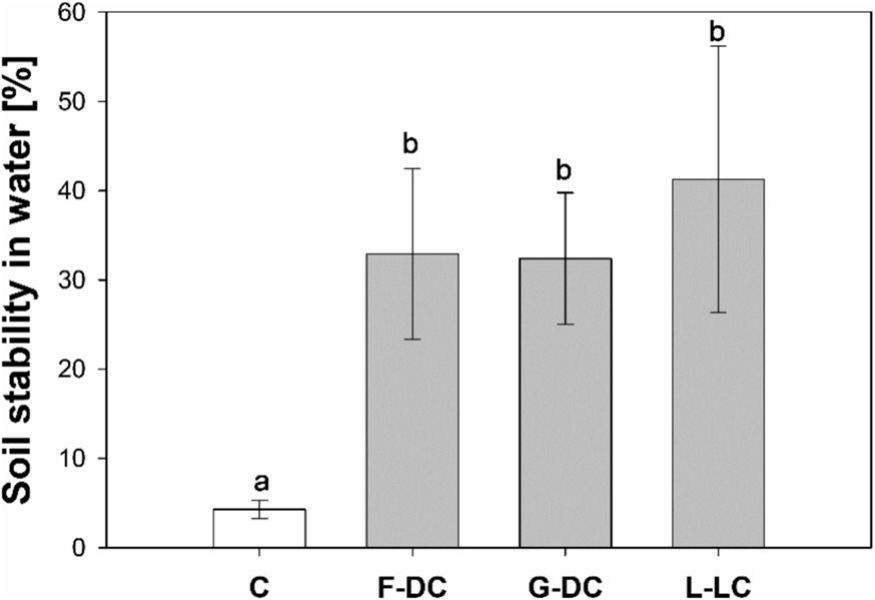
Soil stability in water measured in the soil core sampled from the middle of each longitudinally-divided column (fallow and vegetated soil columns). Means are reported ± standard error of mean (*n* = 5). Letters indicate a statistically significant difference between treatments, as tested using one-way ANOVA followed by post hoc Tukey’s test [log-transformed data, *F-value* = 5.62, *p-value* = 0.008]. Acronyms: fallow control columns [C]; *Daucus carota* [F-DC]; *Deschampsia cespitosa* [G-DC]; *Lotus corniculatus* [L-LC]

The stability of soil cores in water can inform on soil structural development during the twelve months (5-months for establishment + 7-months decomposition treatment) since packing the soil in the columns, as well as the quality of the soil structure. Cores including root channels were 8-times more stable than the control cores (Fig. 11). For instance, whilst less than 7% of the control soil was stable in water, on average 36% of the root-channel soils was stable. In particular, *L. corniculatus* soil showed up to 83% stable structure in water.

## Discussion

This study highlighted that presence and decomposition of roots of different species significantly influence soil hydraulic conductivity, water retention and stability. Plant species and soil depth showed a clear interaction, with different species varying in their ability to influence hydraulic conductivity (*K*_*s*_) down the soil profile. In general, rooted soils showed more uniform *K*_*s*_ values down soil depth, compared with unvegetated soil columns where *K*_*s*_ exponentially reduced with depth. Root decomposition following the development of root channels translated in an abrupt increase in *K*_*s*_ and changes to the *K*_*s*_-depth trends observed in soil with living roots. Moreover, this study provided new data on the distinct physical properties of root-channel soils, which can underpin water flow from preferential flow domain to matrix domain, as well as plant-soil interactions within root channels.

### Root-induced changes to hydraulic conductivity down the soil profile

All species significantly increased *K*_*s*_ compared to unvegetated soil (control; Fig. 1). However, large differences were observed amongst species with minimum and maximum values recorded in the grass *F. ovina* and the legume *L. corniculatus.* In particular, *F. ovina* was unable to significantly alter *K*_*s*_ (Fig. 1)*.* Species-effect on *K*_*s*_ has been discussed by Boldrin et al. (2022) and explained by the differences in root morphology between species. This previous work has clearly demonstrated a negative relation between specific root length and *K*_*s*_-increase induced by roots. Plants with coarse and taproot systems have greater potential to increase water infiltration into the soil, compared with plants with fibrous roots. This difference in root system can explain the consistent increase in *K*_*s*_ under woody vegetation (coarse roots) and the large variability under herbaceous vegetation with root systems mainly represented by fine roots (< 2 mm) (Archer et al. 2013; Ghestem et al. 2011; Leung et al. 2015a). Although the testing of soil columns with different species showed clear differences, these cannot inform the influence of species on *K*_*s*_ down the soil profile. Indeed, the influence of roots on soil properties is generally a function of root depth, with large differences between species (Gregory 2022; Jackson et al. 1996). An increase in *K*_*s*_ might be associated with both the larger values found in shallow soil layers, partially induced by desiccation cracks (Song et al. 2017), and the preferential flow path associated with the root-shoot transition zone, as well as with the *K*_*s*_ increase induced by rhizopores and root channels down the entire soil profile.

Changes in *K*_*s*_ in different layers down the soil column (Fig. 3) provide new data and understanding of *K*_*s*_-depth relation in rooted soils with contrasting species. In the unvegetated columns (control), *K*_*s*_ showed a notable decrease with soil depth (Fig. 3a), with on average 44-times slower water flow at the bottom of the soil column (255–315 mm) compared to the top layer (3–63 mm). This is consistent with the literature on *K*_*s*_ changes down soil depth in unrooted soils (Li et al. 2011; Sun 2018), and generally explained with the overburden pressure increase from the above soil layers and associated porosity changes as depth gradually increases. In our 0.3 m soil columns, dry bulk density generally increased with soil depth (e.g., from 1.31 ± 0.01 g/cm^3^ at 3–63 mm to 1.37 ± 0.02 g/cm^3^ at 255–315 mm, Table 2). This phenomenon could have been induced by the soil-packing in the columns, where pressure was applied to each layer to reach a target volume (i.e., bulk density). Compaction (i.e., bulk density) increase with depth in the top 0.3 m is commonly observed in agricultural fields and earthworks. Moreover, irrigation during the growth period (4 months) might have contributed to the migration of fine silt and clay particles into the deeper soil layers, causing a reduction in pore volume. A similar *K*_*s*_ decrease with soil depth was also observed in the soil columns vegetated with *F. ovina* (Fig. 3d). Indeed, this species showed no significant effects on *K*_*s*_ down the entire soil profile, confirming the observations in the large soil columns (Fig. 1) and previously explained with root-architecture traits (Boldrin et al. 2022). In contrast, *K*_*s*_ in the soil columns vegetated with *D. carota* and *L. corniculatus* showed a distinct and significantly different (compared to the control) relation with depth. In the top layer (3–63 mm) of columns vegetated with these two species, we observed a remarkable decrease in *K*_*s*_ when compared to the control soil. These decreases in *K*_*s*_ can be explained by soil densification induced by the radial growth of *D. carota* and *L. corniculatus* taproots, which have large-diameters in shallow soils, and the consequent loss of porosity in this soil layer. Indeed, both these species show large radial-growth of taproot in shallow soil. These taproots taper and branch into secondary roots in deeper soil layers. Densification of soil close to roots was reported by Bruand et al. (1996), who found that the soil surrounding maize roots had 23% less pore volume, compared to the bulk soil. Given the larger radial-growth and diameter of *D. carota* taproots, compared to maize roots, this phenomenon might have affected a larger volume of soil in our study. Interestingly, *K*_*s*_ in both the *D. carota* and *L. corniculatus*-vegetated soils did not decrease with soil depth, and peaked at the same depth (129–189 mm). Thus, any *K*_*s*_ decrease in the deeper layers (as consistently observed in the control), induced by compaction and pore clogging, was alleviated by the presence of roots. Within agricultural soils, this phenomenon has therefore the potential to reconnect the top- and sub-soil layers previously disconnected by tillage practices, and the development of plough pans. This approach has been suggested in bio-tillage solutions (Zhang and Peng 2021). The grass *D. cespitosa* showed intermediate responses. Whilst we found no *K*_*s*_ changes in the top-soil layer (3–63 mm) of these columns, this species increased *K*_*s*_ in the deeper soil layers (> 192 mm). This can be explained by the biomass and length distribution down the soil profile. Indeed, root biomass and length of *D. cespitosa* in the large soil columns tested for *K*_*s*_ (Fig. 1) were greater in the deeper soil layer (>26 cm depth, Fig. 2). Greater biomass and length in deeper soil layers can be explained by the proliferation of secondary order roots in deeper soil layers.

### Changes in hydraulic conductivity after root decomposition

Vegetation is a dynamic system driven by both the ontogeny of individual plants and ecological succession, where species composition and community structure change over time.

This process implies disappearance of individual plants (i.e., death) and species (i.e., change of species composition). This dynamic opens a fundamental question when plant species are selected to engineer soil structure to deliver functions and services such as erosion control, slope stabilisation and soil–water regulation (e.g., flood management): are we losing soil-functions associated with a given species when plants die and/or are replaced through the process of ecological succession? Of course, the answer can largely vary in relation to the function of our interest and this work mainly focussed on water flow in soil.

The present study clearly demonstrated that root-induced increase in *K*_*s*_ was not only maintained after plant depth but maximised by root decomposition and the development of empty root channels (Figs. 4 and 6). Indeed, after root decomposition (incubation in the heated glasshouse) *K*_*s*_ increased in all vegetated cores with an average increase of 17-times. However, the increase in *K*_*s*_ largely varied with species (Fig. 4), with *D carota*, *L. corniculatus* and *D. cespitosa* showing up to 82, 21 and 14-times increases in *K*_*s*_ respectively compared to the control. The effect of decomposition on *K*_*s*_ was generally greater in the top-soil of columns vegetated with *D. carota* and *L. corniculatus*. In particular, *D. carota* soil-columns showed an abrupt increase in *K*_*s*_ in the top-soil layer (+ 82-times at 3–63 mm), which gradually attenuated moving down the soil column (e.g., + 5-times at 255–315 mm). *D. carota* has a root system composed of a coarse and deep taproot with fewer small lateral roots, whose decomposition results in continuous root-channels that progressively narrow with depth, as can be seen from the CT-scan images and the photos of cores before and after decomposition (Fig. 7 and Suppl. Fig. 3). In contrast, the decomposition of roots from the grass *D. cespitosa* had a small effect on *K*_*s*_ in the top-soil layer and a relatively greater effect in deeper soil layers. This larger *K*_*s*_ increase in deeper soil can be explained by greater *D. cespitosa* root proliferation in deeper soil layers. Indeed, *D. carota* and *D. cespitosa* root systems have opposite trends in terms of both root density and root length density down the soil profile (Fig. 2). Whilst the root system of *D. carota* showed a remarkable decrease in biomass and almost no change in root length with depth, indicating a gradually narrowing primary root with few lateral roots, *D. cespitosa* showed an increase in both biomass and length with depth (Fig. 2). The intermediate effect of the decomposition of the *L. corniculatus* roots can be explained by its dimorphic root system, where the main tap root branches in many thinner secondary roots. Indeed, the root system of *L. corniculatus* showed both a biomass decrease (similar to *D. carota*) and an increase in length (similar to *D. cespitosa*) with depth. As expected, the control soil cores exhibited a decrease in *K*_*s*_ when retested after seven months (Figs. 4 and 6). A decrease in *K*_*s*_ is expected for repeated tests on the same unstructured soil core, because water flow during testing can break down aggregates and clog pores with fine particles transported by seepage.

Observed increases in *K*_*s*_ induced by root decomposition, and the notable differences between species, highlighted how soil structure improvement by time-limited intervention, such as seasonal cover crops or biennial species (e.g., *D. carota*) in agricultural rotations, can have prolonged effects on soil *K*_*s*_ after the disappearance of “soil improver plants”. For instance, cover crops with taproot systems in a no-tillage rotation will provide the greatest impact on soil porosity and *K*_*s*_ during the subsequent crops, which will benefit from low resistance paths for root growth (empty root channels) and water redistribution in deeper soil layers (i.e., reduced water loss by runoff and evaporation). Increasing *K*_*s*_ and hence drainage in agricultural landscapes can reduce waterlogging and runoff erosion in the fields during wet periods as well as mitigate the risk of floods at catchment level (Antolini et al. 2020; Deasy et al. 2014; Wilkinson et al. 2019).

In zero-tillage agriculture, empty root channels are also fundamental to bio-tillage and the alleviation of soil compaction. Indeed, roots prefer growing in existing macropores due to lower mechanical-resistance to penetration and easier access to water and air. For instance, 20% (in top-soil) to 90% (in sub-soil) of wheat (*Triticum aestivum*) roots were found inside existing macropores in a zero-tillage field previously cultivated with *Medicago sativa* (White and Kirkegaard 2010). This role of root channels and other bio-pores becomes fundamental when soil structure is degraded by compaction and the formation of plough pans which disconnect the top- and sub-soils. Continuous root channels, such as the ones observed in *D. carota* columns, can bypass compacted pans and hence reconnect top- and sub-soils. For instance, the dry bulk density of 1.4 g/cm^3^ in our columns would have resulted in a significant mechanical impedance for a cereal crop such as barley (Loades et al. 2013).

The changes in *K*_*s*_ down the soil profile after 7-months incubation in a cold store at 5 °C (Fig. 5) show the effect of root decomposition during winter when decomposition slows down, as well as the potential effects of cold storage of soil cores before *K*_*s*_ testing in the laboratory. The small difference in *K*_*s*_ between the vegetated and unvegetated soils after incubation in the cold store highlights that in the absence of conditions conducive to microbial activity (i.e., temperature), *K*_*s*_ changes after plant death were negligible. Therefore, the observed abrupt increases in *K*_*s*_ after 7-months incubation in the heated glasshouse highlight that to achieve this in the field may take significantly longer than a single season. These results also open questions on laboratory testing of *K*_*s*_ after relatively long-term storage of samples. Although the decomposition-induced changes in *K*_*s*_ were prevented by the low temperature in the cold store, moisture fluctuations were not buffered with consequent development of cracks and shrinkage of the soil. Although evident cracks and shrinkage disappeared after saturation and associated soil swelling, their effects on soil structure and soil adhesion to the acrylic ring might have persisted. Therefore, both temperature and moisture should be controlled when samples are stored before *K*_*s*_ testing, to avoid both biological and physical alterations of soil structure.

Roots of different species and plant functional types vary in relation to their decomposability as a function of lignin (= decreased decomposability) and nitrogen concentrations (= increased decomposability), with graminoids (grasses) generally having more recalcitrant root tissues than eudicots (Prieto et al. 2016; Roumet et al. 2016). Therefore, future work should account for different root decomposability characteristics under field variability. Indeed, while the present study clearly highlights the influence of contrasting species on *K*_*s*_ following root decomposition, further work is necessary to corroborate present results and determine mechanistic relations between root architecture, decomposition and *K*_*s*_ including precise quantification of root architecture before decomposition, different plant functional-types and greater number of replications.

### Physical properties of root-channel soil

The large increase in *K*_*s*_ due to root channel development (Figs. 4 and 6) opens fundamental questions on water relations and water redistribution in the bulk soil surrounding these channels. Physical properties of root channel soils (i.e., channel walls) affect the definition of the boundary conditions and control the transition (water movement) between the “preferential-flow domain” to the “matrix domain”. Furthermore, root-channel properties can affect the root-soil interactions of roots growing in existing empty channels. Results in Figs. 8 –11 provide new data on the physical properties of this distinct part of the soil mass, which may have much greater implications and impacts than its limited volume.

Although the root channels, developed after the decomposition of roots, resulted in a notable increase in *K*_*s*_ down the soil profile (Figs. 4 and 6), water absorption and transmission (i.e., sorptivity) through the soil surrounding the channels were reduced when compared to the bulk soil (control) (Fig. 9).

For instance, whilst *D. carota* root channels induced the greatest increase in *K*_*s*_ down the soil (Fig. 4 b), root-channel soil had significantly lower sorptivity in both the top- and sub-soil (Fig. 9a). This may be explained by a localised reduction in porosity following soil densification and particle reorientation by root-radial growth (Bruand et al. 1996). This finding is consistent with the observed *K*_*s*_ reduction in the *D. carota* top-soil before root decomposition (Fig. 3b), as well as with the generally greater soil hardness (Fig. 8). In light of these findings, we can hypothesise that the large root channels developed after the decomposition of coarse roots can result in rapid water transmission down the soil profile along preferential flow paths, but limited water redistribution in the surrounding soil matrix. Depending on the initial ratio of vertical to horizontal permeability, this may make the rooted soil more or less isotropic in permeability. The decrease in sorptivity in the root channels of *D. cespitosa* and *L. corniculatus* may be explained by the increase in hydrophobicity. This might have been the result of root exudates in the rhizosphere when the root was alive, and organic compounds from root decomposition and associated bacterial processes (Doerr et al. 2000). During root growth and soil penetration, polysaccharides and lipides are exudated at the root tip in the rhizosphere. These exudates can increase water retention in the soil surrounding the roots. However, root exudates become hydrophobic upon drying, causing a zone of water repellent soil in the vicinity of roots or rhizosphere hydrophobicity (Carminati et al. 2010; Kroener et al. 2014; Zeppenfeld et al. 2017). At individual root scale, the hydrophilic – hydrophobic dynamic of root exudates has been explained by hydraulic-buffering of water fluctuations and associated hydraulic stress (Carminati et al. 2010). For instance, hydrophobic exudates in the rhizosphere can prevent fast rewetting and consequent osmotic shock. On a wider “plant-community” scale, Zeppenfeld et al. (2017) hypothesised that rhizosphere hydrophobicity can provide an advantage to deep-rooted plants in the competition for water by limiting the water availability to shallow-rooted plants. Root exudates can vary in their ability to induce soil hydrophobicity. For instance, Naveed et al. (2018) reported greater hydrophobicity in sandy loam soil treated with *Zea mays* root-exudates compared with the same soil treated with *Hordeum vulgare* exudates. However, no effect was reported for clay loam soil. Thus, the observed decrease in sorptivity in the root channels can be explained by two independent physical and chemical mechanisms: (i) soil densification by radial root growth (e.g., *D. carota*; Figs.8 and9) and (ii) soil hydrophobicity induced by root exudates and decomposition (e.g., *D. cespitosa*; Fig. 9).

The observed changes in water retention in the soil of root channels (Fig. 10) are consistent with previous works on rhizosphere and rooted soils (Bengough 2012; Boldrin et al. 2022; Leung et al. 2015b; Whalley et al. 2013). For example, Leung et al. (2015b) reported an increase in water content for a given matric suction in rooted soils, similar to our results for *D. carota* root-channels (Fig.10). However, it should be noted that this and other similar studies have been based on soil samples permeated by roots, and hence the results are often explained by root occupation of pore spaces (i.e., decrease in pore diameter and volume). Ng et al. (2016) developed a SWRC model for rooted soils based on a “fourth soil volumetric-phase” given by root volume in soil. A further model by Ni et al. (2019) was developed to capture both the effects of root growth and root decay on SWRCs and *K*_*s*_. In contrast, the results of the present study (Fig. 10) are based on the soil surrounding empty root channels. Therefore, root occupancy of pore volume cannot explain the observed alteration of soil water retention curves in the present study. In the soil samples cored from the root channels, the increase in water content for a given matric suction can be explained by a localised increase in soil organic matter following root decomposition, reorganisation of soil structure by root growth and root exudate release during the establishment period (Lal 2020; Whalley 2005). The greater water holding capacity of the root-channel soils can potentially be beneficial to roots growing in these empty pores.

Root-channel soils had a significantly greater stability in water compared to the control bulk soil (Fig. 11). Therefore, according to thresholds given in Bartlova et al. (2015), the soil structural quality moved from “very low” to “medium” following root growth and decomposition. An increase in aggregate stability has been associated with root intrusion (Boldrin et al. 2022; Tisdall and Oades 1979) and increase in organic matter (Tisdall and Oades 1982). However, in the present study, we can exclude any direct root mechanical reinforcement because the root material was almost completely decomposed. Increased stability in water is important when root-channel functions such as *K*_*s*_ are of interest. Indeed, poor soil stability in water would quickly translate into soil dispersion in water and consequent clogging of channels. Therefore, soil hydrological functions should always be considered in conjunction with structural stability and integrity to avoid misleading conclusions, especially in structural unstable soils. Finally, it is important to note that interpretations on physio-chemical alterations of root-channel walls, such as densification and organic-deposits, are based on observed patterns and literature, rather than conclusive results on mechanistic processes. Therefore, future work is necessary to quantify physical alteration (e.g., soil-density changes), as well chemical alteration (e.g., the nature of organic compounds release during root decomposition) in soil surrounding the root-channels.

## Conclusions

This study investigated the hydrological impacts of root channels accounting for variables including species, soil depth and root decomposition. Furthermore, to the best of our knowledge, this is the first study exploring the physical properties of soil surrounding root-channels remaining after root decomposition. Rooted soil showed more uniform and greater saturated hydraulic conductivity (*K*_*s*_) down the soil depth compared with control soil, where *K*_*s*_ exponentially reduced with depth. Root decomposition and subsequent development of root channels resulted in an abrupt increase in *K*_*s*_ and changes in the distribution of *K*_*s*_ with depth, compared to soils with living roots. Despite these increases in *K*_*s*_, water sorptivity through the soil surrounding the channels (i.e., walls of the channel) showed a decrease when compared to the control soil. Therefore, we can hypothesise that the large root channels developed after the decomposition of coarse roots can result in fast preferential water flow down the soil profile, with limited water redistribution into the surrounding soil matrix. This decrease in sorptivity had different causes in relation to plant species, including soil densification in *D. carota* (forb) and soil hydrophobicity in *D. cespitosa* (grass). While the observations in the present study clearly highlight the influence of root decomposition on water flow within the soil, further work is necessary to determine mechanistic relations between root architecture, decomposition and soil hydraulic properties, including precise quantification of root architecture before decomposition, different plant functional-types and greater number of replications. Moreover, it is important to note that this study was based on repacked soil in columns under controlled environmental conditions. Further work is needed to understand the role of temperature and water fluctuations in the development of root channels. For instance, what is the necessary time for the development of root channels functional to bio-tillage in zero-tillage agriculture under different climates and bulk densities (i.e., mechanical impedance)? When do these benefits become effective after the clearing of cover-crops and the subsequent root decomposition? Furthermore, under field conditions large pore spaces are quickly occupied by new root systems. Future work should account for system dynamics under both variable abiotic and biotic factors.

## Supplementary information

Below is the link to the electronic supplementary material.

Fig. 12 Diagram and photo of soil column and its layers (five soil cores). See Hydraulic conductivity down vegetated soil section in Materials and Methods(PNG 546 KB)

ESM 1  (TIF 855 KB)

Fig. 13 (PNG 541 KB)

ESM 2 (TIF 683 KB)

Fig. 14 (PNG 1.84 MB)

ESM 3 (TIF 1.03 MB)

ESM 4 (DOCX 18.3 KB)

## Data Availability

The data that support the findings of this study are available from the corresponding author David Boldrin [david.boldrin@hutton.ac.uk], upon reasonable request
