# Supplementary figures and images for: Root decomposition affects soil hydraulic properties in four contrasting herbaceous species
Source: Plant Soil. 2026 Feb 16;520(2):1387–407. doi: 10.1007/s11104-026-08331-y (PMC13065577; doi:10.1007/s11104-026-08331-y)

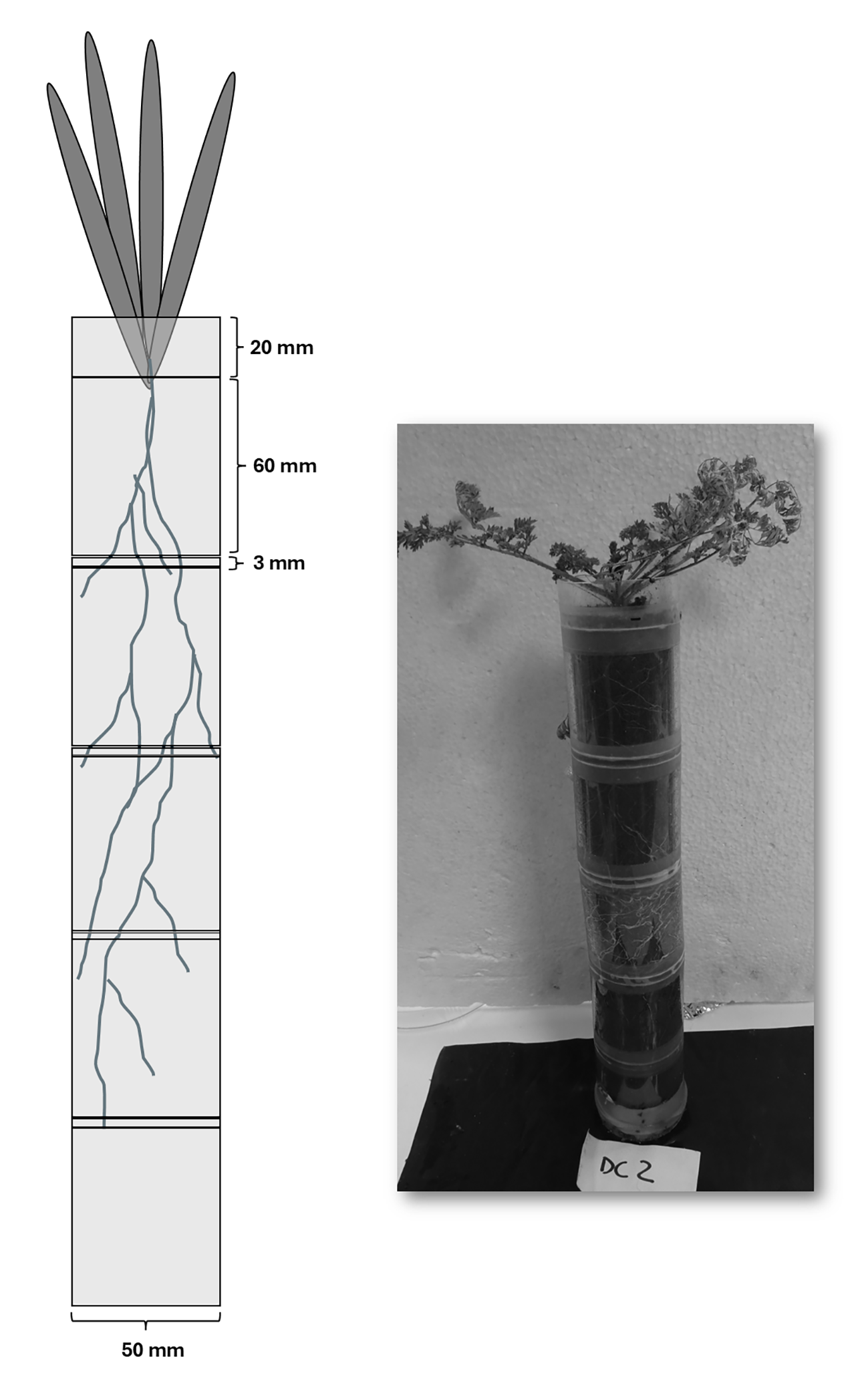

Supplement: Supplementary file 1 — Diagram and photo of soil column and its layers (five soil cores). See Hydraulic conductivity down vegetated soil section in Materials and Methods(PNG 546 KB) [file 11104_2026_8331_Fig12_ESM.png]

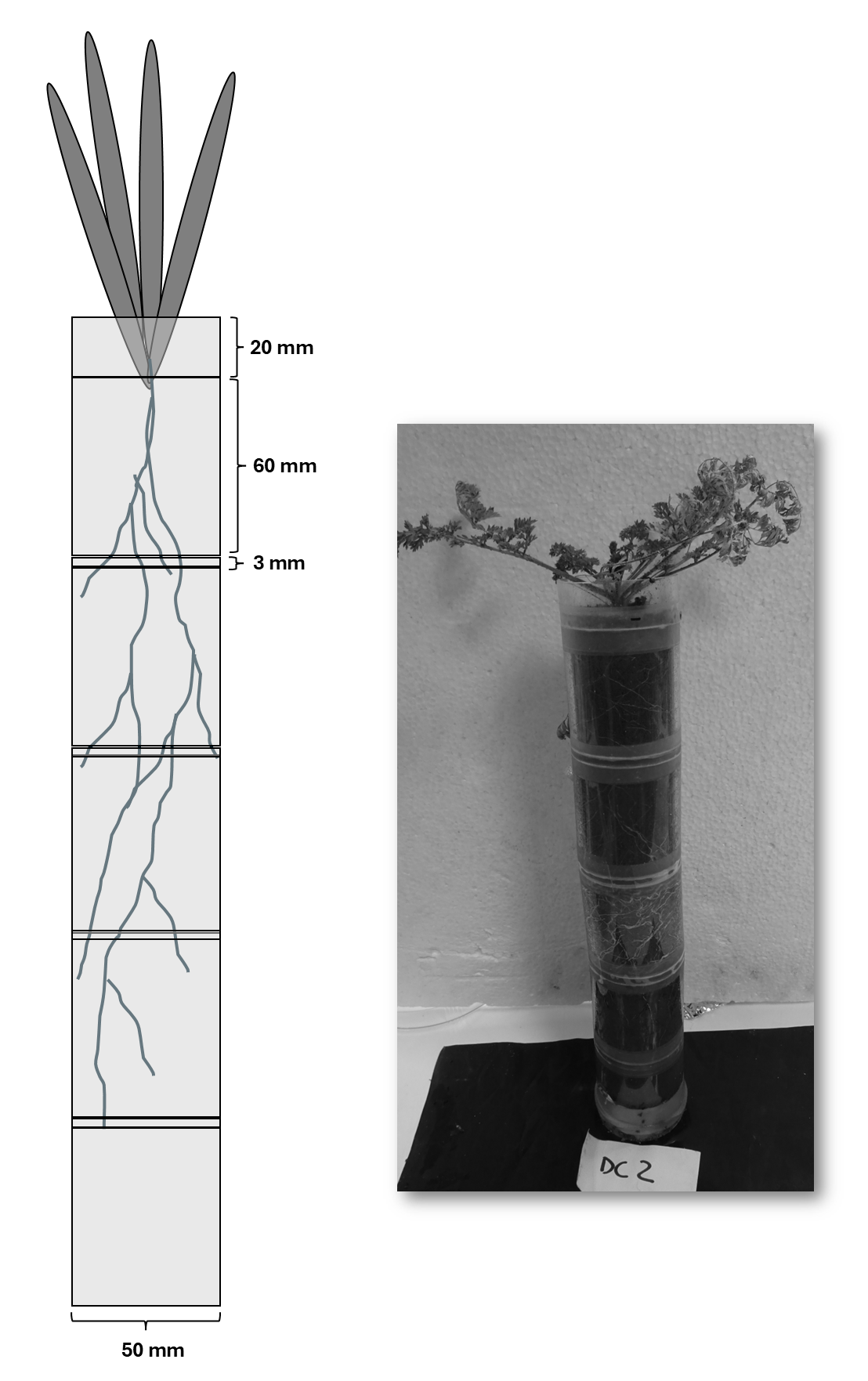

Supplement: Supplementary file 2 — (TIF 855 KB) [file 11104_2026_8331_MOESM1_ESM.tif]

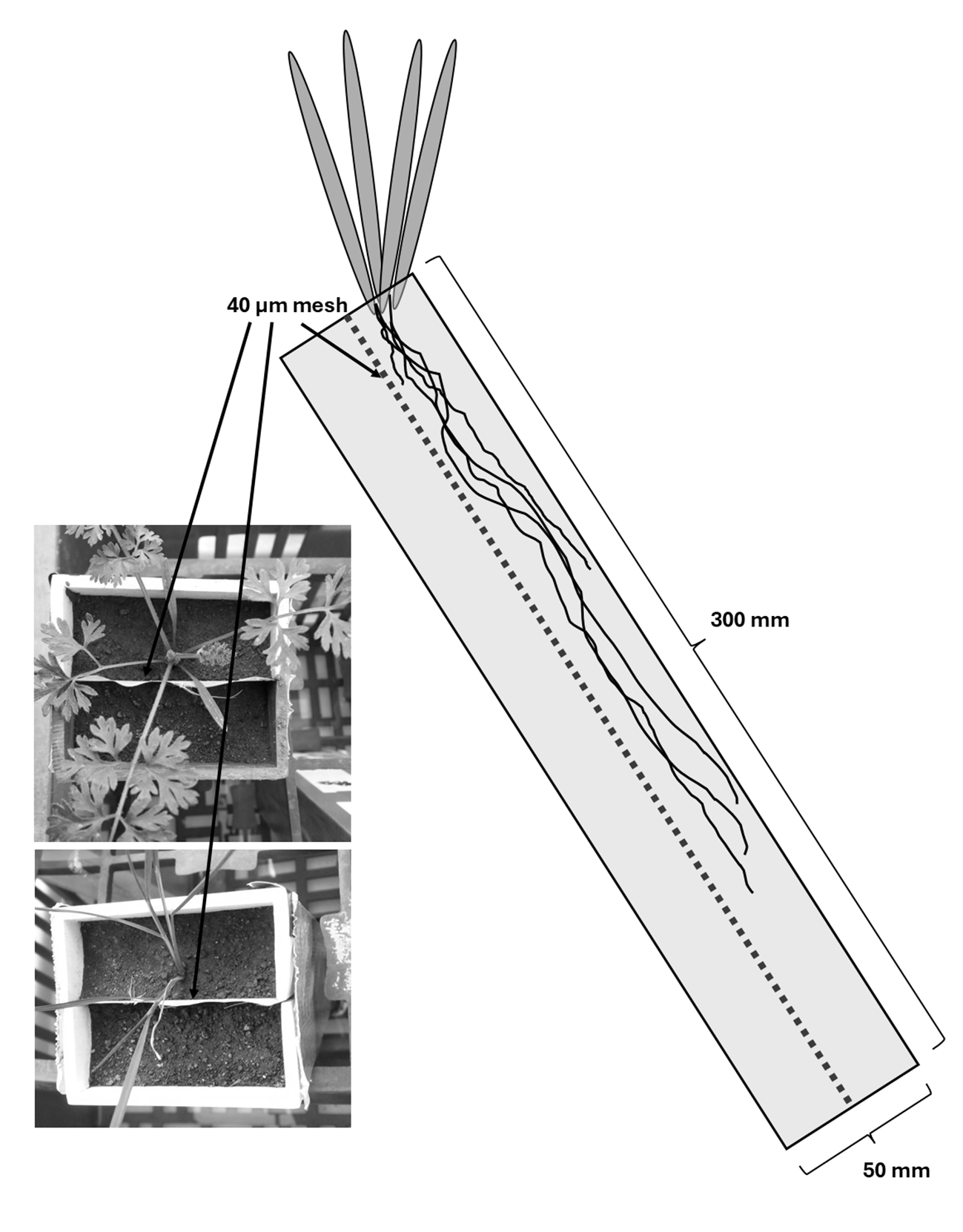

Supplement: Supplementary file 3 — (PNG 541 KB) [file 11104_2026_8331_Fig13_ESM.png]

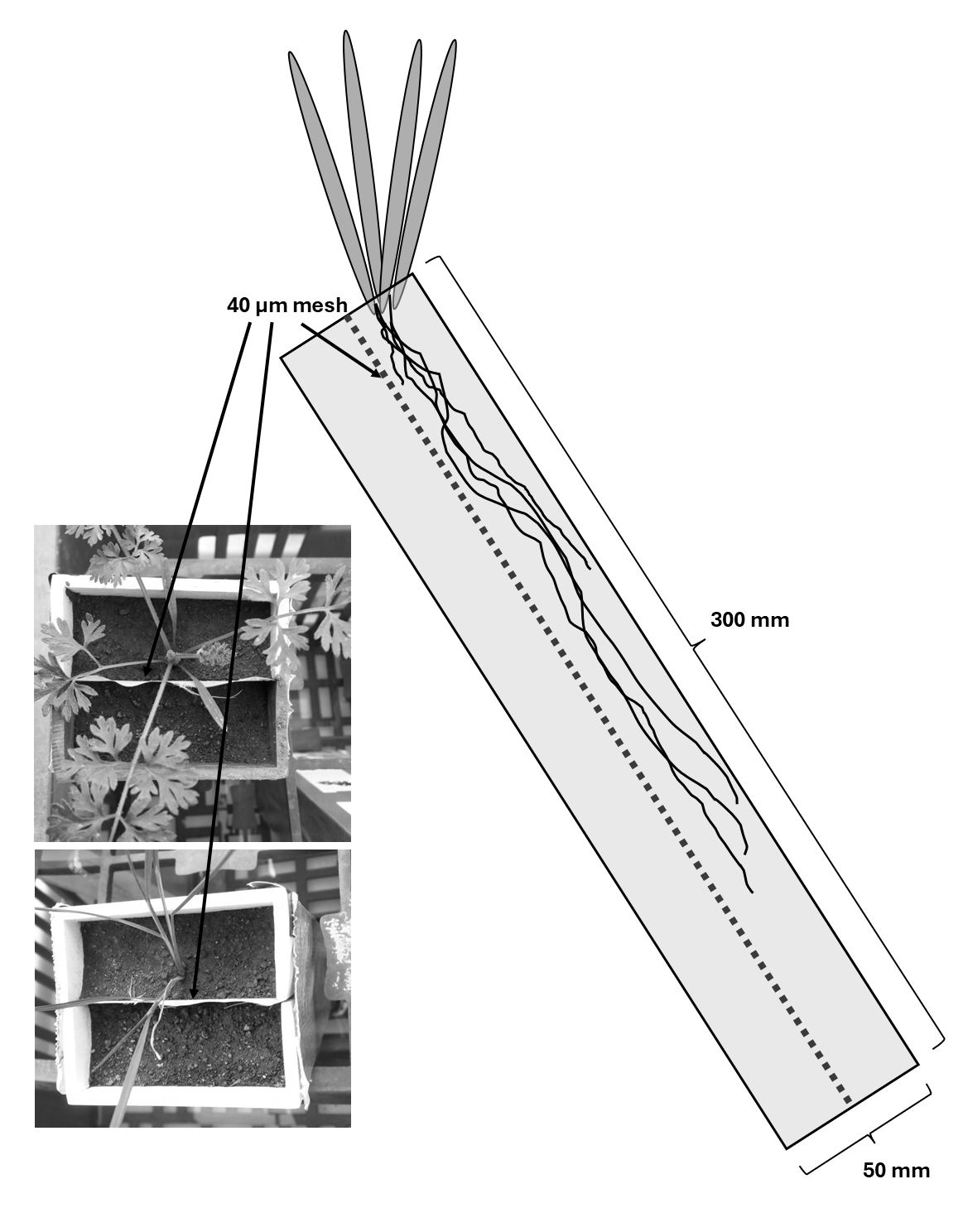

Supplement: Supplementary file 4 — (TIF 683 KB) [file 11104_2026_8331_MOESM2_ESM.tif]

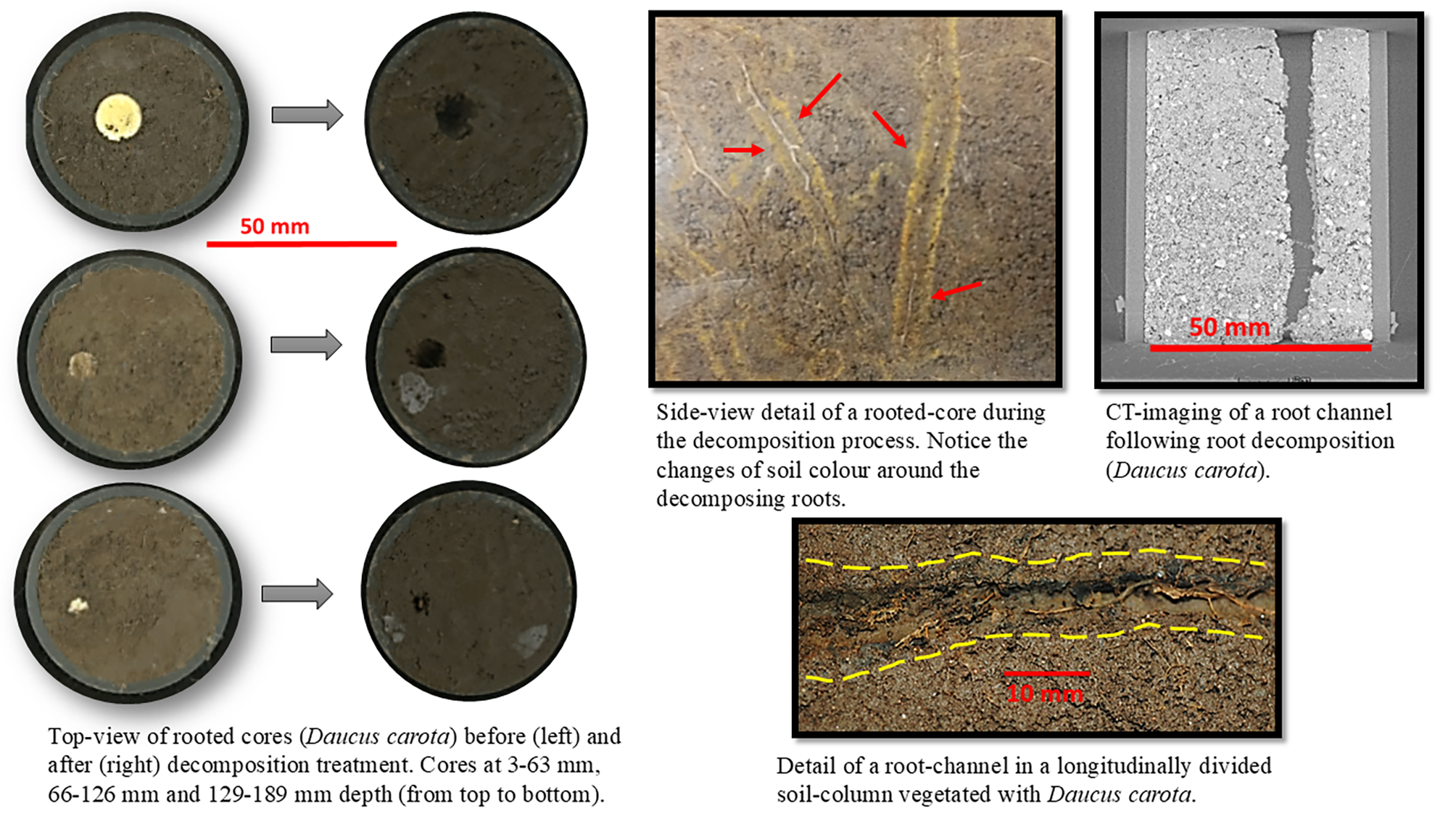

Supplement: Supplementary file 5 — (PNG 1.84 MB) [file 11104_2026_8331_Fig14_ESM.png]

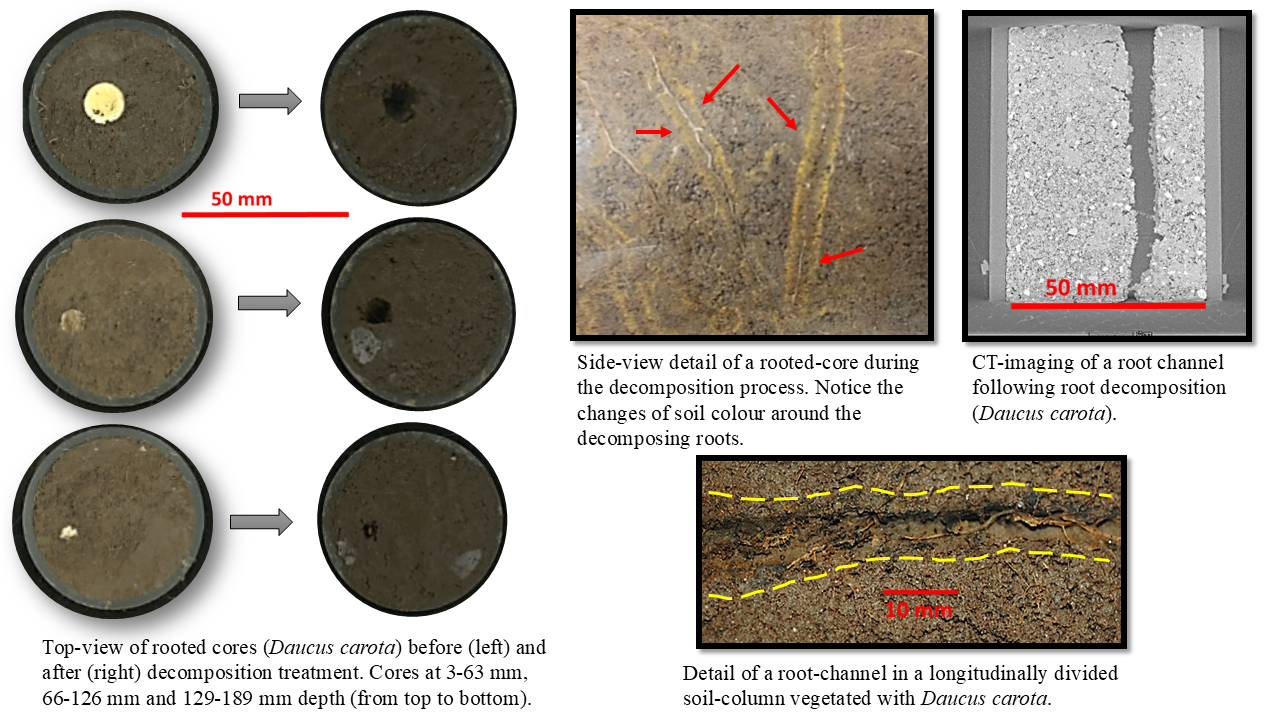

Supplement: Supplementary file 6 — (TIF 1.03 MB) [file 11104_2026_8331_MOESM3_ESM.tif]
