# Supplementary material for: Root decomposition affects soil hydraulic properties in four contrasting herbaceous species
Source: Plant Soil. 2026 Feb 16;520(2):1387–407. doi: 10.1007/s11104-026-08331-y (PMC13065577; doi:10.1007/s11104-026-08331-y)
Supplement: Supplementary file 7 — (DOCX 18.3 KB) [file 11104_2026_8331_MOESM4_ESM.docx]

**Suppl. Table 1**

Statistical analysis of results in Fig 2. Data tested using one-way ANOVA followed by post hoc Tukey’s test for statistical differences (Capital letters) between treatments at the same soil depth [Top table] and statistical differences (Lowercase letters) between layers in the same treatment [Bottom table]. Acronyms: fallow control columns [C]; *Daucus carota* [F-DC]; *Deschampsia cespitosa* [G-DC]; *Lotus corniculatus* [L-LC].

| Depth, mm | *C* | *F-DC* | *G-DC* | *G-FO* | *L-LC* | *Data-transformation* | *F-value* | *P-value* |
| --- | --- | --- | --- | --- | --- | --- | --- | --- |
| 3 - 63 | *B* | *A* | *AB* | *AB* | *AB* | Log | 3.44 | 0.037 |
| 66 - 126 | *A* | *A* | *A* | *A* | *A* | Log | 0.56 | 0.695 |
| 129 - 189 | *A* | *AB* | *AB* | *A* | *B* | Log | 4.36 | 0.017 |
| 192 - 252 | *A* | *B* | *B* | *AB* | *AB* | Log | 6.01 | 0.005 |
| 255 - 315 | *AB* | *C* | *BC* | *A* | *C* | Log | 9.23 | <0.001 |

| Treatments | 3 - 63 mm | 66 - 126 mm | 129 - 189 mm | 192 - 252 mm | 255- 315 mm | *Data-transf.* | *F-value* | *P-value* |
| --- | --- | --- | --- | --- | --- | --- | --- | --- |
| *C* | *b* | *ab* | *a* | *a* | *a* | Log | 8.85 | <0.001 |
| *F-DC* | *a* | *a* | *a* | *a* | *a* | Log | 0.37 | 0.827 |
| *G-DC* | *b* | *ab* | *ab* | *ab* | *b* | Log | 3.69 | 0.028 |
| *G-FO* | *c* | *bc* | *abc* | *ab* | *a* | Log | 6.58 | 0.007 |
| *L-LC* | *a* | *a* | *a* | *a* | *a* | Log | 2.53 | 0.084 |
